# Description of a fossil camelid from the Pleistocene of Argentina, and a cladistic analysis of the Camelinae

**DOI:** 10.1186/s13358-020-00208-6

**Published:** 2020-10-07

**Authors:** Sinéad Lynch, Marcelo R. Sánchez-Villagra, Ana Balcarcel

**Affiliations:** Palaeontological Institute and Museum, University of Zurich, Karl-Schmid-Strasse 4, 8006 Zurich, Switzerland

**Keywords:** Lamini, Camelinae, South America, North America, Phylogeny

## Abstract

We describe a well-preserved South American Lamini partial skeleton (PIMUZ A/V 4165) from the Ensenadan (~ 1.95–1.77 to 0.4 Mya) of Argentina. The specimen is comprised of a nearly complete skull and mandible with full tooth rows, multiple elements of anterior and posterior limbs, and a scapula. We tested this specimen’s phylogenetic position and hypothesized it to be more closely related to *Lama guanicoe* and *Vicugna vicugna* than to *Hemiauchenia paradoxa*. We formulate a hypothesis for the placement of PIMUZ A/V 4165 within Camelinae in a cladistic analysis based on craniomandibular and dental characters and propose that future systematic studies consider this specimen as representing a new species. For the first time in a morphological phylogeny, we code terminal taxa at the species level for the following genera: *Camelops*, *Aepycamelus, Pleiolama*, *Procamelus,* and *Alforjas.* Our results indicate a divergence between Lamini and Camelini predating the Barstovian (16 Mya)*. Camelops* appears as monophyletic within the Camelini. *Alforjas taylori* falls out as a basal member of Camelinae—neither as a Lamini nor Camelini. *Pleiolama* is polyphyletic, with *Pleiolama vera* as a basal Lamini and *Pleiolama mckennai* in a more nested position within the Lamini. *Aepycamelus* and *Procamelus* are respectively polyphyletic and paraphyletic. Together, they are part of a group of North American Lamini from the Miocene epoch.

**Electronic supplementary material:**

The online version of this article (10.1186/s13358-020-00208-6) contains supplementary material, which is available to authorized users.

## Introduction

Exceptional fossils are key to solving taxonomic and phylogenetic questions given the rich and reliable anatomy they preserve. Some of them are still waiting in the field, while some are in museum collections in which proper curation and care allow multiple researchers to re-assess them. We present a case of the latter, with the study of specimen PIMUZ A/V 4165 from Barranca del Parana, San Nicolas (Buenos Aires province, Argentina) (Roth 1889; Schulthess 1920), part of a collection assembled by the celebrated Swiss-Argentinian paleontologist Santiago Roth, 1850–1924 (Bond 1999). PIMUZ A/V 4165 was initially identified as a *Palaeolama* (Roth 1889; Schulthess 1920)*,* a genus of the ‘tribe’ Lamini. Its stratigraphic age is Pampeano inferior (Roth 1889), also known as the Ensenadan (Cione et al. 2015). We describe this material for the first time and test its position in a cladistic analysis that also addresses other aspects of camelid evolution in the Americas.

The Camelidae appeared during the middle Eocene in North America (Honey et al. 1998). *Poebrotherium wilsoni,* one of its earliest members, is recorded from the late Eocene through the early Oligocene. According to Honey et al. (1998), the ‘family’ Camelidae underwent four radiation events. The ‘sub-family’ Protolabinae appeared in the third radiation, which took place in the late Oligocene and early Miocene. The ‘sub-family’ Camelinae, which includes the ‘tribes’ Lamini and Camelini, appeared between the late Hemingfordian and early Barstovian (~ 17.5–14 Mya), during the fourth radiation.

Honey et al. (1998) estimated a divergence between the Lamini and Camelini during the Barstovian (~ 16–12 Mya). However, the timing of this split is still ambiguous, partly due to systematically problematic taxa, including the genus *Aepycamelus*. Honey et al. (1998) attributed *Aepycamelus* (late Hemingfordian to late Hemphillian, ~ 17.5–6 Mya) to the Lamini, while observing that members of this genus could also be ancestral to both Lamini and Camelini. Adding to the discussion, studies based on mitochondrial data estimated the divergence of Lamini and Camelini to approximately 25 Mya, during the Arikareean (Cui et al. 2007).

**Fig. 1:**
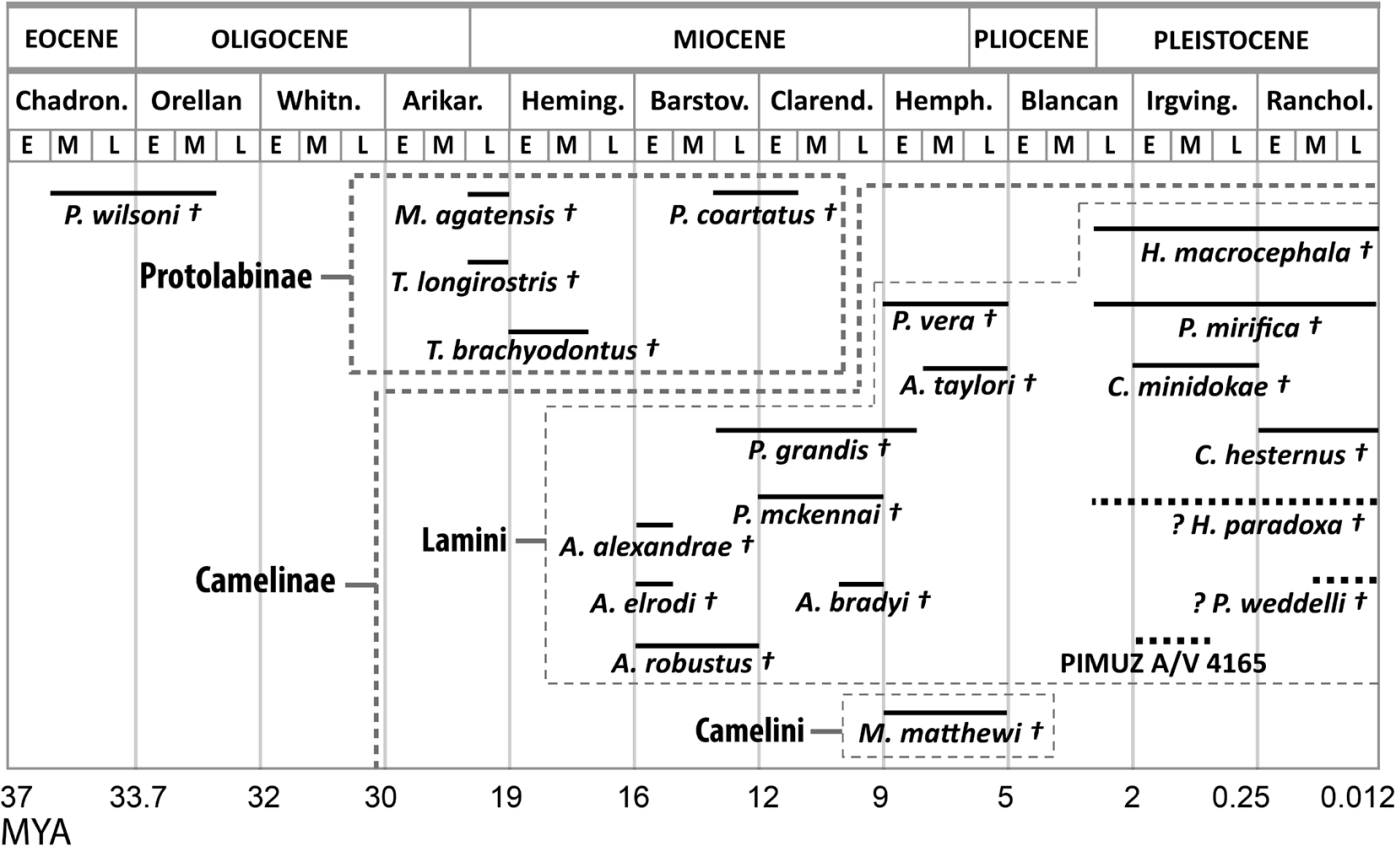
Time range of extinct species included in this study. Not to scale, species time range approximate. Species positioning in the Protolabinae follows Honey (1998) and Honey (2007). Species positioning in the Camelinae, Lamini, and Camelini follow our results. Species range for North American species following Baskin and Thomas (2016); Bravo-Cuevas and Jiménez-Hidalgo (2015); Harrison (1979); Honey and al. (1998); Honey and Taylor (1978); Pagnac (2005); Peterson (1911); Prothero (2005); Webb and Meachen (2004). Absolute ages for North American land mammal age following Woodburne (2004). Species range for South American species are uncertain and discussed by Scherer (2013). Absolute ages for South American land mammal age following Verzi et al. (2004); Soibelzon and al. (2008); Cione and Tonni (2001); Cione and Tonni (2005); Cione et al. (2015). Abbreviations: Chadron. = Chadronian; Whitn. = Whitneyan; Arikar. = Arikareean; Heming. = Hemingfordian; Barstov. = Barstovian; Clarend. = Clarendonian; Hemph. = Hemphillian; Irving. = Irvingtonian; Ranchol. = Rancholabrean; E = early; M = middle; L = late

The generic diversity of camelids decreased during the late Miocene when *Aepycamelus* and the last members of the Protolabinae are last recorded (Honey et al. 1998). The last North American camelids disappeared in the late Pleistocene (Kurtén and Anderson 1980) (Fig. 1).

### Previous studies on camelids

One of the first morphology-based phylogenies including a broad range of camelids was that of Webb (1965), which also provided an osteological description of *Camelops*. Honey and Taylor (1978) later revised the relationships among Protolabinae. In 1979, Harrison described the genus *Alforjas* and suggested a phylogeny for the Camelinae, later refining it with the inclusion of North American extinct giant Camelini (Harrison 1979, 1985). A few years later, Honey et al. (1998) proposed an extensive taxonomic revision of the entire Camelidae clade. They attributed the following extinct genera: *Aepycamelus*, *Blancocamelus*, *Hemiauchenia*, *Palaeolama, Alforjas, Camelops,* and *Pliauchenia,* to the Lamini and *Procamelus*, *Megatylopus*, *Titanotylopus*, *Megacamelus* and *Gigantocamelus* to the Camelini.

Webb and Meachen (2004) later invalidated the genus *Pliauchenia* and re-positioned some of its members within a new genus, *Pleiolama,* along with two newly-described species: *Pleiolama mckennai and Pleiolama vera.* In the same work, they also described *Alforjas magnifontis*. Scherer (2013) proposed the most recent phylogeny based on a cladistic analysis of a broad range of South American species and new postcranial characters. She considered the following South American taxa as valid: *Hemiauchenia paradoxa*, *Palaeolama major*, *Palaeolama weddelli*, *Lama guanicoe*, *Lama castelnaudi*, *Vicugna*, *Vicugna provicugna,* and *Eulamaops parallelus*.

Baskin and Thomas (2016) validated two *Camelops* species: *C. hesternus* and *C. minidokae*. Morphological phylogenies have predominantly assigned *Camelops* to the Lamini clade (Harrison 1979; Honey et al. 1998; Scherer 2013), but recently novel proteomic (Buckley et al. 2019) and genetic (Heintzman et al. 2015) studies indicated a closer connection between this genus and the Camelini. By contrast, no progress has been made to determine the intra- and inter-generic affinities of *Aepycamelus*. Morphological phylogenies have also neglected the intra-generic relationships of *Alforjas*, *Pleiolama, Procamelus,* and *Camelops*. Our study provides novel insights into these areas of the Camelid tree, based on revision of original materials in museums, a critical assessment of the literature, and an analysis of these data.

## Materials and methods

### Materials

Specimen PIMUZ A/V 4165 is from the Roth Collection at the Palaeontological Museum of the University of Zurich. It was collected in Barranca del Parana, San Nicolas (Buenos Aires province, Argentina) (Roth 1889; Schulthess 1920). The stratigraphic age is Ensenadan (early to middle Pleistocene) (Roth 1889; Schulthess 1920; Cione et al. 2015), which dates approximately from ~ 1.95–1.77 to 0.4 Mya (Verzi et al. 2004; Soibelzon et al. 2008). To investigate the taxonomic and systematic allocation of this fossil, we conducted a phylogenetic analysis of several South and North American taxa. We used *Poebrotherium wilsoni*, a basal camelid (Honey et al. 1998), as the outgroup. For the ingroup, we included members of the Camelinae (see Additional files 1, 2). Several North American taxa (*Pleiolama*, *Camelops, Aepycamelus, Procamelus,* and *Alforjas*), previously scored as genera by Scherer (2013), Honey et al. (1998) and Harrison (1979, 1985), are now scored as species. For *Pleiolama* and *Camelops*, we included all species currently accepted. We also included four species *of Aepycamelus* and a single one of the genus *Alforjas*. All Protolabinae genera (*Michenia*, *Tanymykter*, *Protolabis*) are also in the ingroup.

Most of the terminal taxa are scored from a single, well-preserved specimen. A few taxa, such as *Camelops minidokae*, *Megatylopus matthewi,* and *Palaeolama mirifica,* were scored with more fragmentary material. For *Lama* and *Vicugna*, we included only the wild forms (*L. guanicoe*, *V. vicugna*). For *Camelus*, we scored material from the domestic *C. bactrianus*.

### Characters

**Fig. 2:**
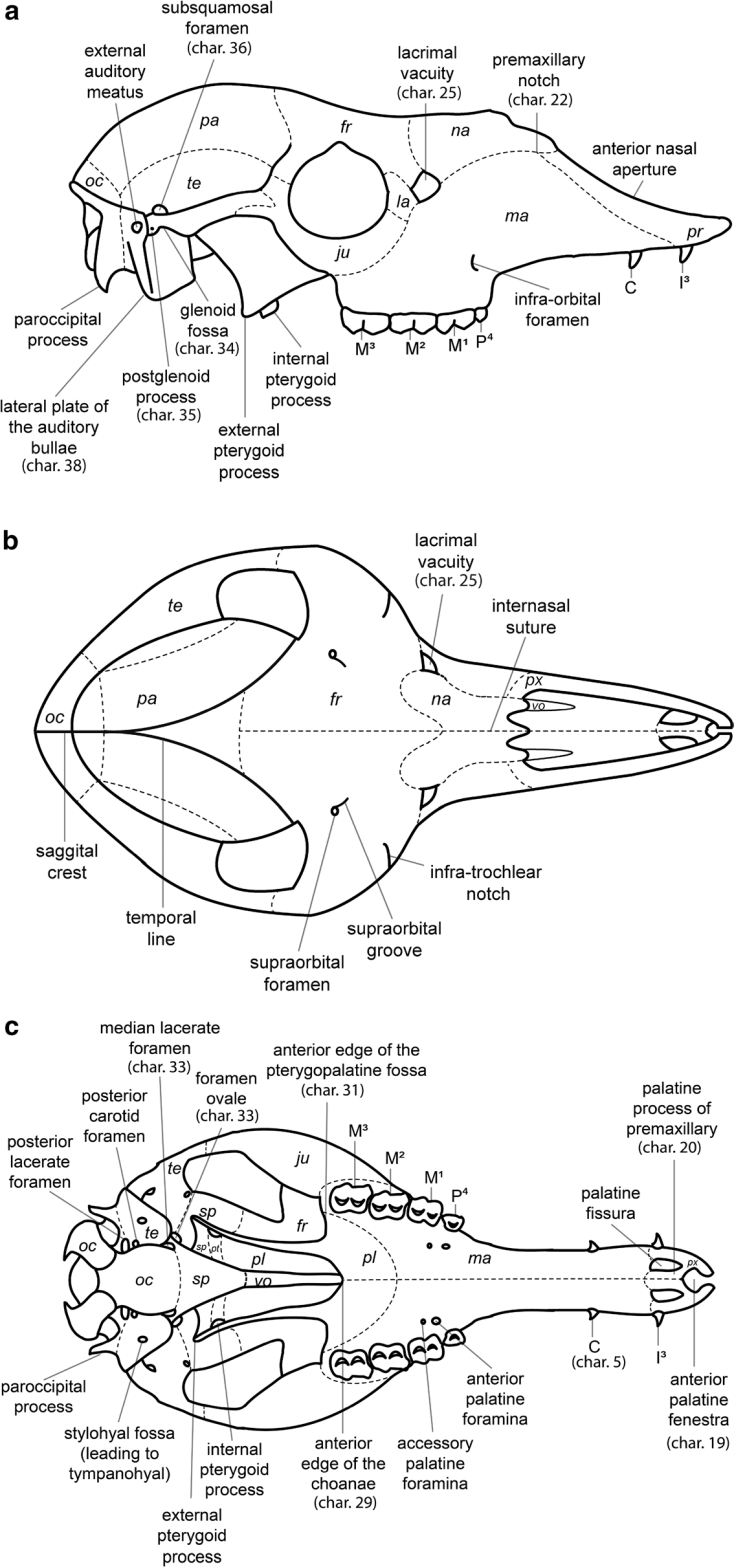

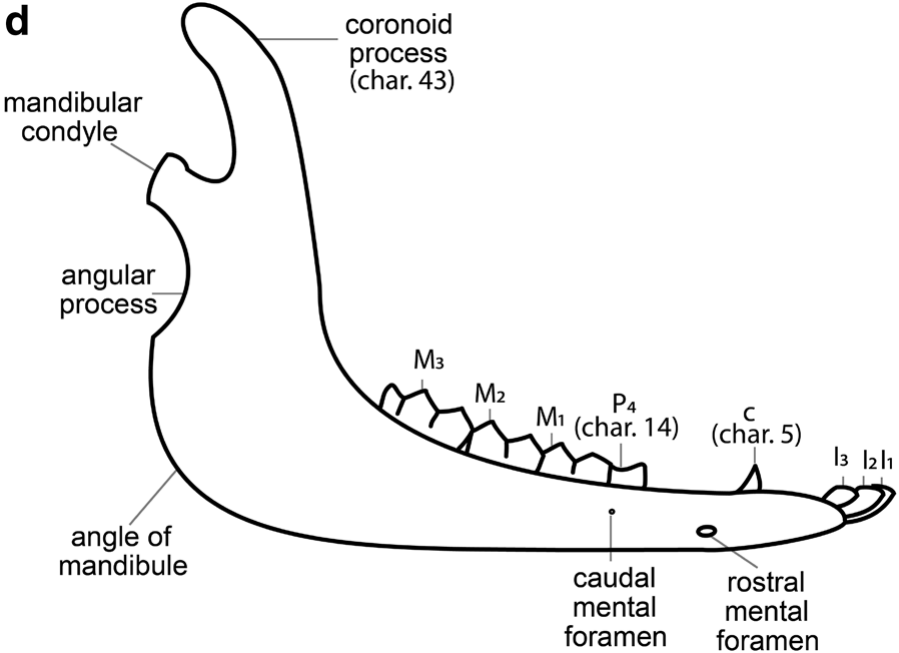
Schematic drawings of *L. guanicoe*.** a** Skull, lateral view.** b** Skull, dorsal view.** c** Skull, occlusal view. **d** Mandible, lateral view. Abbreviations: pr = premaxillary bone; ma = maxillary bone; fr = frontal bone; la = lacrimal bone; ju = jugal bone; te = temporal bone; oc = occipital bone; sp = sphenoid bone; pt = pterygoid bone; vo = vomer bone; pl = palatine bone; pa = parietal bone; I^3^ = third upper incisor; I_1_ = first lower incisor; I_2_ = second lower incisor; I_3_ = third lower incisor; C = Upper canine; c = Lower canine; P^1^ = first upper premolar; P_1_ = first lower premolar; P_2_ = second lower premolar; P^4^ = fourth upper premolar; P_4_ = fourth lower premolar; M^1^ = first upper molar; M_1_ = first lower molar; M^2^ = second upper molar; M_2_ = second lower molar; M^3^ = third upper molar; M_3_ = third lower molar

**Fig. 3:**
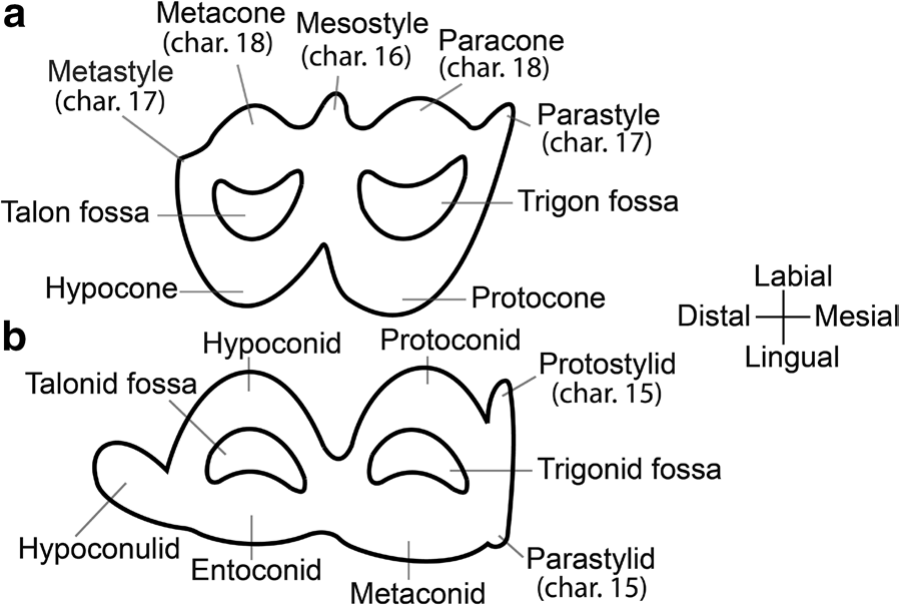
Schematic drawings of *L. guanicoe*’s teeth. **a** M^3^, occlusal view.** b** M_3_, occlusal view

We concentrated on craniomandibular characters exclusively; no postcranial characters were included, since many taxa in the analysis had no (or only fragmentary) postcranial elements. Terminology for skull and mandible morphology follows Webb (1965), Pacheco Torres et al. (1986) and El Allali et al. (2017) (Fig. 2). Dental terminology follows Hershkovitz (1982) (Fig. 3).

We reviewed previous systematic analyses by Scherer (2013), Honey et al. (1998), Harrison (1979, 1985), and Webb (1965). Characters were updated and adapted, as noted. Five characters, not previously used in cladistic analyses, were based on information provided by Webb (1965) and by Honey (2007). In total, we present 17 new characters. We measured the hypsodonty index as per Shockey (1997). We found it to vary greatly, particularly in our sample of *V. vicugna*, likely due to differences in the level of wear. Therefore, we omitted this character from our matrix. We followed the scoring of Scherer (2009, 2013) for P^3^ (char. 12) and P_3_ (char. 13) in *L. guanicoe* and *V. vicugna,* as she had access to more material from these species and because their states were ambiguous in our sample.

For ratio characters (chars. 44–49), we defined states by gap-coding (figures of the measurements are in Additional file 3). A detailed list of the characters included in this study is presented below.

### Dental characters

1. First upper incisor (I^1^): *present (0); absent (1)*. (Ch.10, Scherer (2013); ch.1, Harrison (1979); Honey et al. (1998); Harrison (1985); Honey and Taylor (1978)).
2. Second upper incisor (I^2^): *present (0); absent (1)*. (Ch.11, Scherer (2013); mod. ch.2, Harrison (1979); Honey et al. (1998); Harrison (1985); Honey and Taylor (1978)).
3. Enamel layer on the lingual side of lower incisors (I_1-3_): *thick (0); thin or absent (1)*. (Modified from ch.12, Scherer (2013); Honey et al. (1998); Harrison (1985)).
4. Crown of lower incisors (I_1-3_): *spatulated (0); cylindrical (1)*. (Modified from ch.12, Scherer (2013); Honey et al. (1998); Harrison (1985)).
5. Upper and lower canine transverse section (C, c): *rounded (0); laterally compressed (1)*. (Modified from ch.13, Scherer (2013); mod. ch.4 + 5, Harrison (1979); Honey et al. (1998); Harrison (1985)).
6. Upper canine position (C): *close to third incisor (I*^*3*^*) (*< *1 cm) (0); distant to third incisor (I*^*3*^*) (≥ 1 cm) (1).* (Distance from the distal border of the third incisor to the mesial border of canine).
7. Lower canine position (c): *close to incisor (I*_*3*_*) (*< *1 cm) (0); far from incisor (I*_*3*_*) (≥ 1 cm) (1).* (Distance from the distal border of the third incisor to the mesial border of canine). (Fig. 4).

**Fig. 4:**
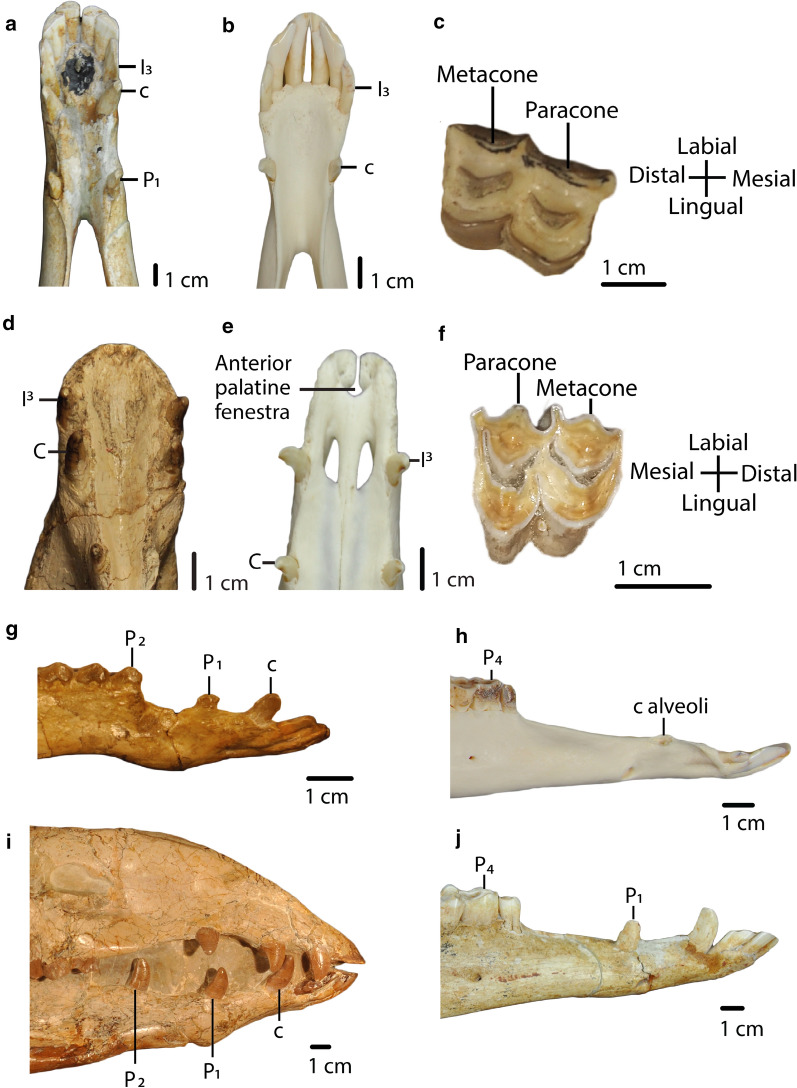
**a**,** b**: Char. 7: “Lower canine position (c)”. **a** *H. macrocephala* (UF 205,750), scored close from incisor (I_3_) (< 1 cm) (0). **b** *L. guanicoe* (ZM 17,209), scored far from incisor (I_3_) (≥ 1 cm) (1). **c**, **f**: Char. 18: “Metacone and paracone on upper molars (M^1^-M^3^)”. **c** Right M^2^, *P. coartatus* (AMNH 73,377), scored weakly developed (0). **f** Left M^2^, *P. weddelli* (PUN 1), scored well developed (1). **d**, **e**: Char. 19: “Anterior palatine fenestra”. **d** *P. coartatus* (AMNH 73,438), ventral view, scored absent (0). **e** *L. guanicoe* (ZM 17,209), ventral view, scored present (1). **g**, **h**, **i**, **j**: Char. 9: “First lower premolar (P_1_)”. **g** *P. wilsoni* (AMNH 47,130), right side, scored premolariform (0). **h** *L. guanicoe* (ZM 17,967), left side, scored absent (2). **i** *A. robustus* (OMNH 016,560), right side, scored caniniform (1). **j** *H. macrocephala* (UF 205,750), right side, scored caniniform (1). **g**, **h**, **i**, **j**: Char. 11: “Second lower premolar (P_2_)”. **g** *P. wilsoni* (AMNH 47,130), right side, scored premolariform (0). **h** *L. guanicoe* (ZM 17,967), left side, scored absent (2). **i** *A. robustus* (OMNH 016,560), right side, scored caniniform (1). **j** *H. macrocephala* (UF 205,750), right side, scored absent (2)
8. First upper premolar (P^1^): *premolariform (0); caniniform (1); absent (2)*. (Modified from ch. 14, Scherer (2013); mod. ch.6, Harrison (1979); mod. ch.“P1 and P/1”, Webb (1965, p. 46); Honey et al. (1998); Harrison (1985)).
9. First lower premolar (P_1_): *premolariform (0); caniniform (1); absent (2)*. (Modified from ch.15, Scherer (2013); mod. ch.7, Harrison (1979); mod. ch.“P1 and P/1”, Webb (1965, p. 46); Honey et al. (1998); Harrison (1985); Fig. 4).
10. Second upper premolar (P^2^): *present (0); absent (1)*. (Ch.16, Scherer (2013); mod. ch.8, Harrison (1979); Modified from ch.“P2 and P/2”, Webb (1965, p. 46); Honey et al. (1998); Harrison (1985); Honey and Taylor (1978)).
11. Second lower premolar (P_2_): *premolariform (0); caniniform (1); absent (2)*. (Modified from ch.17, Scherer (2013); mod. ch.9, Harrison (1979); mod. ch.“P2 and P/2”, Webb (1965, p.46); Honey et al. (1998); Harrison (1985); Honey and Taylor (1978); Fig. 4).
12. Third upper premolar (P^3^): *present (0); absent (1).* (Ch.10, Harrison (1979); ch.18, Scherer (2013); Honey et al. (1998)).
13. Third lower premolar (P_3_): *present (0); absent (1).* (Ch.11, Harrison (1979); ch.19, Scherer (2013); Honey et al. (1998); Harrison (1985)).
14. Fourth lower premolar shape (P_4_): *triangular, with fossetid only on the distal lobe (0); quadrangular, with fossetids on the mesial and distal lobes (1)*. (Ch.20, Scherer (2013)).
15. Protostylids and parastylids (“llama buttresses”) on lower molars (M_1_–M_3_): *small or absent (0); greatly developed (1)*. (Ch.24, Scherer (2013); mod. ch.12, Harrison (1979); mod. ch.“Lower Molars”, Webb (1965, p. 46); Honey et al. (1998); Harrison (1985), see Scherer (2009, Fig. 20, p. 90)).
16. Labial lophids on lower molars (M_1_-M_3_) in occlusal view: *U-shaped or rounded (0); triangular (1)*. (Mod ch.21, Scherer (2013), see Scherer (2009, Fig. 20, p. 90); see Scherer et al. (2007, Fig. 4, p. 41)).
17. Labial styles (“ribs”) on upper molars (M^1^–M^3^): *weakly developed (0); well-developed (1)*. (Mod ch.22, Scherer (2013), see Scherer (2009, Fig. 20, p. 90)).
18. Metacone and paracone on upper molars (M^1^-M^3^): *weakly developed (0); well-developed (1).* (Fig. 4

### Cranial characters

19. Anterior palatine fenestra: *absent (0); present (1).* (Based on Honey (2007); Fig. 4.
20. Position of the most posterior part of the palatine process of premaxillary: *closer to the canine (0); closer to the third incisor (1).* (Fig. 5)

**Fig. 5:**
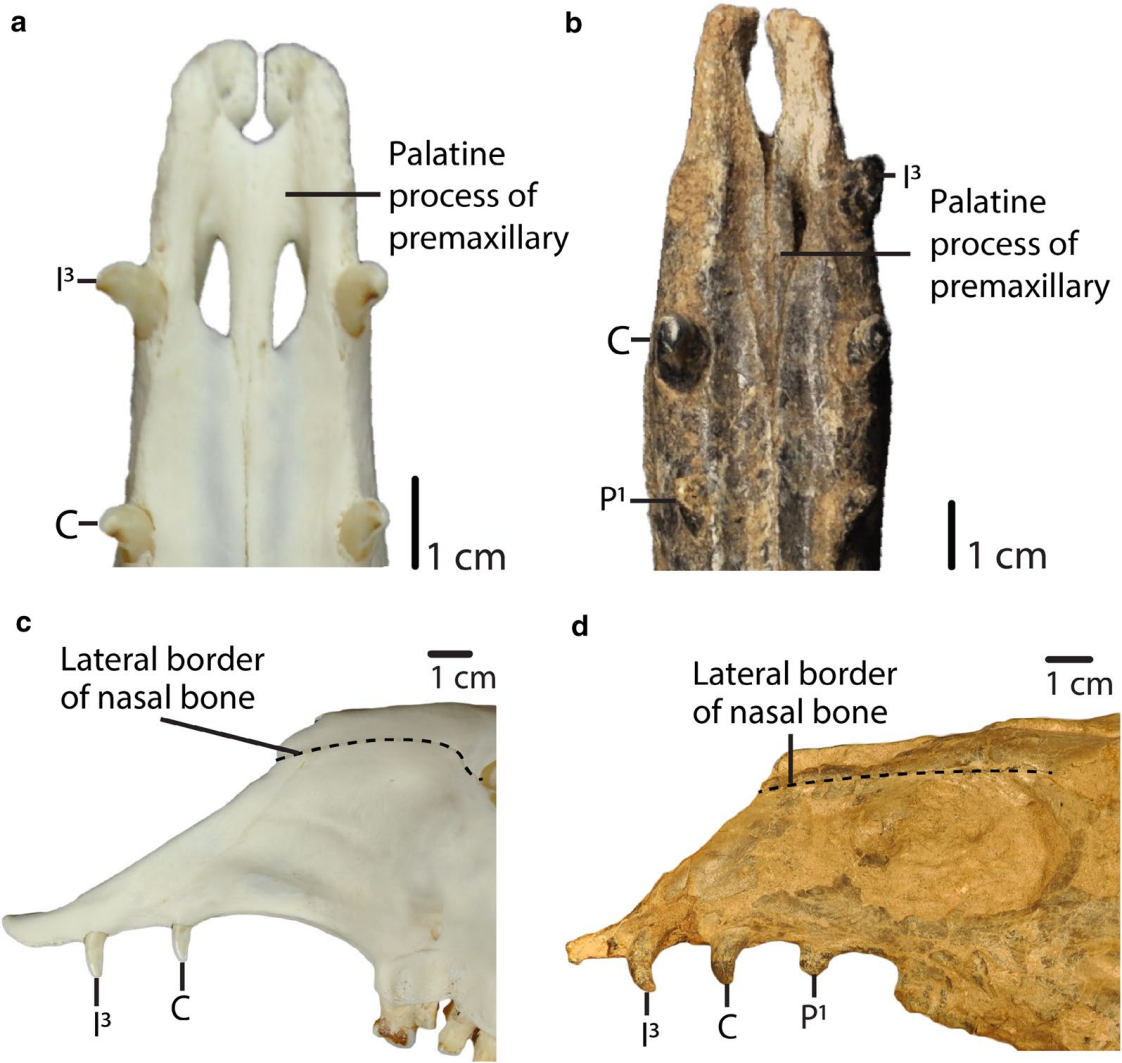
**a**, **b**: Char. 20: “Position of the most posterior part of the palatine process of premaxillary”. **a** *L. guanicoe* (ZM 17,209), scored closer to the third incisor (1). **b** *A. taylori* (AMNH 40,821), scored closer to the canine (0). **c**, **d**: Char. 24: “Lateral border of nasals”. **c** *L. guanicoe* (ZM 17,209), left side, scored curved (1). **d** *T. brachydontus* (AMNH 36,594), left side, scored straight (0)
21. Distance between third incisors compared to the distance between canines (distances on the lingual borders): *distance between I3s greater than the distance between Cs (0); distance between Cs greater than the distance between I3s (1)*. (Modified from Honey and Taylor (1978)).
22. Premaxillary notch: *present (0); absent (1).* (Based on Webb (1965)).
23. Shape of the anterior end of nasal bones in transverse section: *dorso-ventrally flattened (0); arched dorsally (1)*. (Modified from ch.3, Scherer (2013); mod. ch.18, Harrison (1979, Fig. 3, p.11); Honey et al. (1998); Harrison (1985)).
24. Lateral border of nasal bones: *straight (0); curved (1).* (Based on Webb (1965); Fig. 5).
25. Lacrimal vacuity: *large, bordered by four bones (frontal, maxillary, lacrimal, nasal) (0); small, bordered by four bones (frontal, maxillary, lacrimal, nasal) (1); absent (2); bordered by three bones (frontal, maxillary, lacrimal) (3); bordered by four bones (frontal, maxillary, lacrimal, nasal), lacrimal border greatly reduced, frontal border greatly enlarged (4); bordered by two bones (frontal, maxillary) (5).* (Modified from ch.1, Scherer (2013); mod. ch.16, Harrison (1979); Honey et al. (1998); Harrison (1985); Fig. 6).

**Fig. 6:**
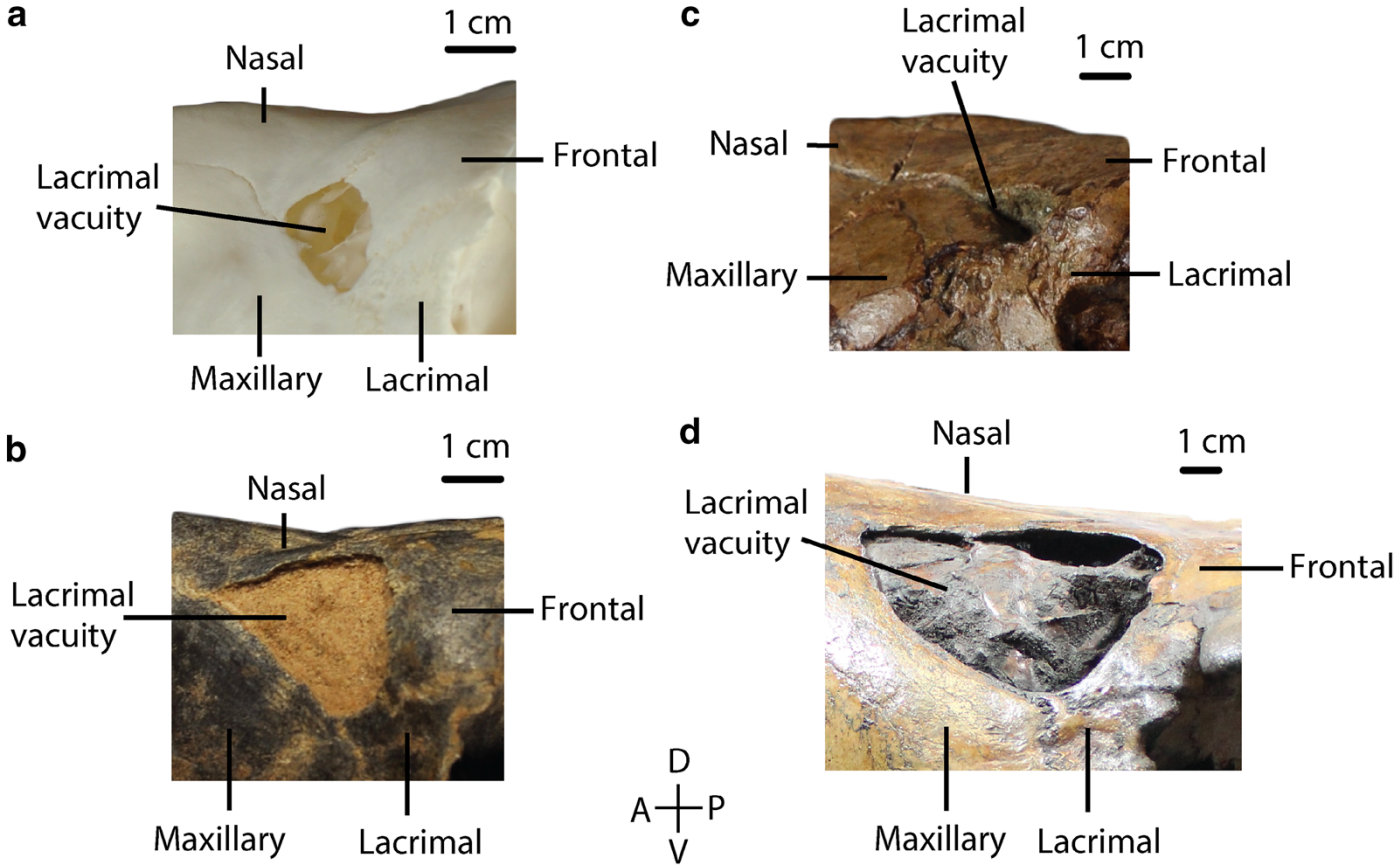
Char. 25: Bones bordering the lacrimal vacuity. **a** *L. guanicoe* (ZM 17,209), left side, scored small, bordered by four bones (frontal, maxillary, lacrimal, nasal) (1) **b** *P. vera* (AMNH 24,670), left side, scored large, bordered by four bones (frontal, maxillary, lacrimal, nasal) (0). **c** *A. alexandrae* (UCMP 26,015), left side, scored bordered by three bones (frontal, maxillary, and lacrimal) (3). **d** *C. hesternus* (UCMP 20,040), left side, scored bordered by four bones (frontal, maxillary, lacrimal, and nasal), lacrimal border greatly reduced, frontal border greatly enlarged (4)
26. Maxillary fossa: *well-developed, large pocket (0); well-developed, small pocket (1); shallow or absent (2)*. (Modified from ch.2, Scherer (2013); mod. ch.“Maxillary Fossa”, p.46, Webb (1965); mod. ch.17, Harrison (1979); Honey et al. (1998); Harrison (1985); Fig. 7).

**Fig. 7:**
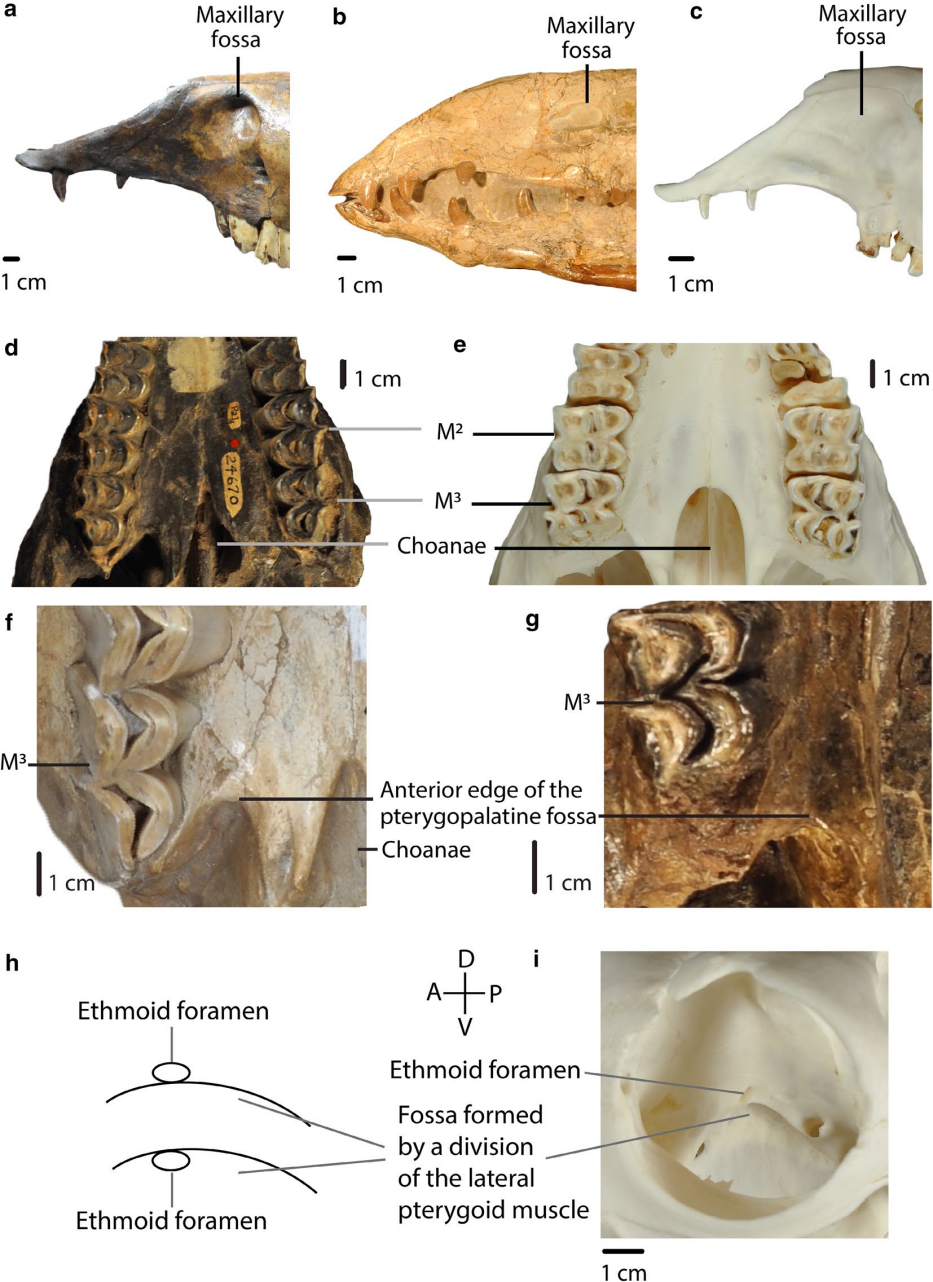
A, B, C: Char. 26: “Maxillary fossa”. **a** *C. hesternus* (UCMP 20,040), left side, scored well-developed, large pocket (0). **b** *A. robustus* (OMNH 016,560), right side, scored well-developed, small pocket (1). **c** *L. guanicoe* (ZM 17,209), left side, scored shallow or absent (2). **d**, **e**: Char. 30: “Shape of the anterior edge of the choanae”. **d** *P. vera* (AMNH 24,670), ventral view, scored V-shaped (0). **e** *L. guanicoe* (ZM 17,209), ventral view, scored U-shaped (1). **f**, **g**: Char. 31: “Anterior edge of the pterygopalatine fossa”. **f** *M. matthewi* (UCMP 31,100), scored at the level of M3 (1). **g** *T. longirostris* (CM 2498), scored posterior to M3 (0). **h**, **i**: Char. 32: “Position of the ethmoid foramen”. **h** Upper: above the fossa formed by a division of the lateral pterygoid muscle (0). Lower: in the fossa formed by a division of the lateral pterygoid muscle (1). **i** *L. guanicoe* (ZM 17,209), right side, scored above the fossa formed by a division of the lateral pterygoid muscle (0)
27. Zygomatic arch in lateral view: *curved (0); straight (1).* (Ch.4, Scherer (2013); ch.20, Harrison (1979); mod. ch.“Zygoma”, Webb (1965, p.46); Harrison (1985)).
28. Orbital process of palatine: *present (0); narrow or absent (1).* (Based on Webb (1965)).
29. Anterior edge of the choanae: *posterior to M*^*3*^* (0), at the level of M*^*3*^* or between M*^*3*^* and M*^*2*^* (1); at the level of M*^*2*^* or between M*^*2*^* and M*^*1*^* (2)*. (Modified from ch.7, Scherer (2013), see Scherer (2009, Fig. 20, p.90)).
30. Shape of the anterior edge of the choanae: *V-shaped (0); U-shaped (1)*. (Fig. 7).
31. Anterior edge of the pterygopalatine fossa: *posterior to M*^*3*^* (0); at the level of M*^*3*^* (1).* (Fig. 7)
32. Position of the ethmoid foramen: *above the fossa formed by a division of the lateral pterygoid muscle (0); in the fossa formed by a division of the lateral pterygoid muscle (1).* (Based on Webb (1965)). (Fig. 7)

### Basicranial characters

33. Foramen ovale and median lacerate foramen: *completely separated by a thick portion of the alisphenoid (0); completely separated by a thin spine of the alisphenoid (1); confluent (2).* (Modified from ch.“Median Lacerate Foramen”, Webb (1965, p. 47)).
34. Position of the glenoid fossa: *close to the ventral surface of basisphenoid (0); well above the ventral surface of basisphenoid (1)*. (Modified from ch.“Glenoid Fossa”, Webb (1965, p. 46)).
35. Postglenoid process and postglenoid foramen: *small-to-absent (0); large (1)*. (Modified from ch.22, Harrison (1979); mod. ch.“Postglenoid Process”, Webb (1965, p. 47); Fig. 8).

**Fig. 8:**
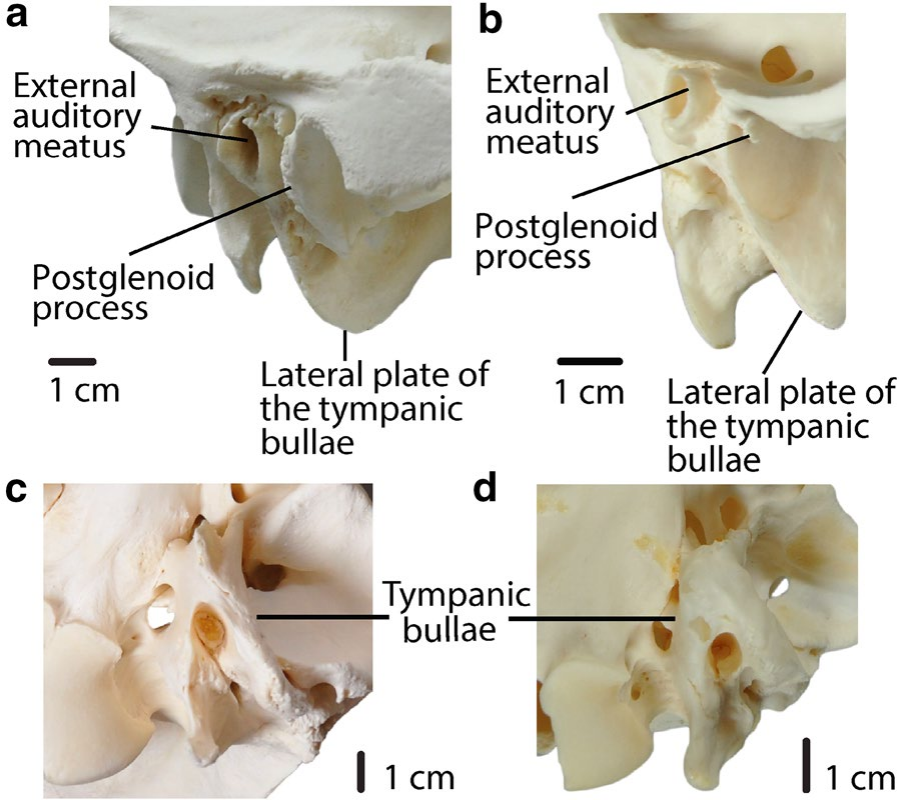
**a**, **b**: Char. 35: “Postglenoid process and postglenoid foramen”. **a** *C. bactrianus* (ZM 17,970), right side, scored large (1). **b** *L. guanicoe* (ZM 17,209), left side, scored small-to-absent (0). **a**, **b**: Char. 38: “Position of the lateral plate of the bullae in lateral view”. **a** *C. bactrianus* (ZM 17,970), right side, scored below external auditory meatus (0). **b** *L. guanicoe* (ZM 17,209), left side, scored anterior to external auditory meatus (1). **c**, **d**: Char. 37: “Tympanic bullae”. **c** *C. bactrianus* (ZM 17,970), ventral view, scored little inflated (1). **d** *L. guanicoe* (ZM 17,209), ventral view, scored greatly inflated (0)
36. Subsquamosal foramina: *small (0); large (1)*. (Modified from ch.“Temporal canal”, Webb (1965, p. 47)).
37. Tympanic bullae: *greatly inflated (0); little inflated (1).* (Based on Webb (1965)) (Fig. 8).
38. Position of the lateral plate of the bullae in lateral view: *below external auditory meatus (0); anterior to external auditory meatus (1)*. (Fig. 8).

### Dentary characters

39. Anterior part of the mandible in lateral view: *straight (0); angled dorsally (1).* (Fig. 9).

**Fig. 9:**
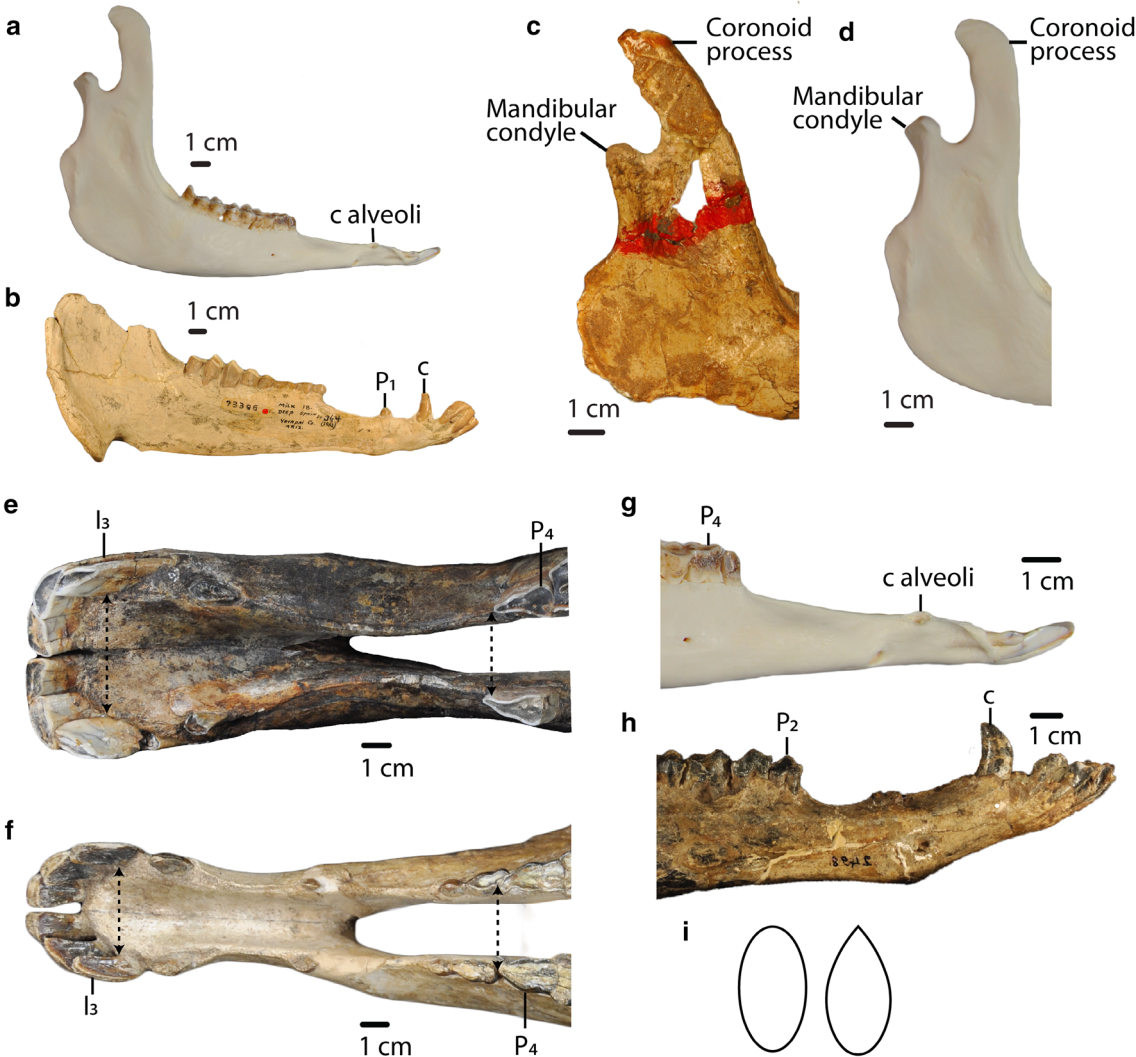
**a**, **b**: Char. 42: “Angle of the mandible in lateral view”. **a** *L. guanicoe* (ZM 17,967), left side, scored rounded (2). **b** *P. coartatus* (AMNH 73,306), right side, scored ventrally produced with lateral flare (1). **c**, **d**: Char. 43: *“*Coronoid process at the level of the mandibular condyle in lateral view”. **c** *T. brachyodontus* (AMNH 36,594), right side, scored angled posteriorly (0). **d** *L. guanicoe* (ZM 17,967), left side, scored straight (1). **e**, **f**: Char. 40: “Distance between mesial end of the third lower incisors compared to the distance between the mesial end of fourth lower premolars”. **e** *C. minidokae* (UCMP 38,448), scored distance between I_3_ greater than distance between P_4_ (1). **f** *P. grandis* (UCMP 32,864), scored distance between P_4_ greater or equal to distance between I_3_ (0). **g**, **h**: Char. 39: “Anterior part of the mandible in lateral view”. **g** *L. guanicoe* (ZM 17,967), left side, scored straight (0). **h** *T. longirostris* (CM 2498), left side, scored angled dorsally (1). **i** Char. 41: “Portion of the mandibular ramus below the mandibular diastema in transverse section”. Left: Little narrowing dorsally, ovoid (1). Right: Strongly narrowing dorsally, tear-drop shaped (0)
40. Distance between mesial end of the third lower incisors compared to the distance between the mesial end of fourth lower premolars: *distance between P*_*4*_* greater or equal to distance between I*_*3*_* (0); distance between I*_*3*_* greater than distance between P*_*4*_* (1).* (Fig. 9).
41. Portion of the mandibular ramus below the mandibular diastema in transverse section: *strongly narrowing dorsally, tear-drop shaped (0); little narrowing dorsally, ovoid (1).* (Fig. 9).
42. Angle of the mandible in lateral view: *posteriorly projected (0); ventrally produced with lateral flare (1); rounded (2).* (Modified from Honey et al. (1998); Honey and Taylor (1978), Fig. 9).
43. Coronoid process at the level of the mandibular condyle in lateral view: *angled posteriorly (0); straight (1).* (Fig. 9).
  *Ratio characters* (see Additional files 3 and 4).
44. Rostral length (ratio between rostral length and length of the skull): *ratio higher than 0.56 (0); ratio lower than 0.56 (1).*
45. Retraction of nasals (ratio between the internasal suture and rostral length): *ratio higher than 0.6 (0); retracted ratio from 0.6 to 0.57(1); ratio lower than 0.57 (2).*
46. Orbit size (ratio between orbital width and length of the skull): *ratio higher than 0.138 (0); ratio lower or equal to 0.138 (1).*
47. Minimum postcanine width (ratio between the minimum postcanine width and width of the skull): *ratio higher then 0.155 (0); ratio from 0.155 to 0.125 (1); ratio from 0.125 to 0.055 (2); ratio lower than 0.055 (3).*
48. Length of mandibular symphysis (ratio between length of the symphysis and length of the mandible): *ratio higher than 0.255 (0); ratio lower than 0.255 (1).*
49. Height of the mandible (ratio between height of the mandible and length of the mandible): *ratio higher than 0.58 (0); ratio lower than 0.58 (1).*

### Systematic analysis

The character matrix is available in the Electronic Supplementary Material (Additional file 5). It comprises 49 morphological characters. Eight of these characters (chars. 8, 9, 11, 26, 29, 33, 45, and 47) were ordered*.* For the ingroup, we examined 18 species and 3 specimens identified at the genus level distributed among 13 Camelinae genera and 3 Protolabinae genera (Additional file 2).

We performed a cladistic analysis using parsimony with a traditional search in TNT (Goloboff and Catalano 2016). We ran 6000 replicates with 10 trees saved per replication. Bremer supports were obtained with suboptimal trees by 7 steps. Bootstraps values were obtained with a cutoff of 50.

## Description of PIMUZ A/V 4165

### Locality and horizon

Specimen PIMUZ A/V 4165 was collected near the Parana river in the locality of San Nicolas (Barranca del Parana, San Nicolas, province of Buenos Aires, Argentina) (see Roth 1888, table 23; Voglino 2008, Fig. 1). There is no further information on the specimen or its precise stratigraphic position within las Barrancas. Geological age is Ensenadan (Early-to-Middle Pleistocene) according to museum archives and current curatorial staff at Museo de La Plata (Roth 1889; Schulthess 1920; Cione et al. 2015, A. Carlini, personal communication, July 18, 2020). However, there has been no modern revision of the stratigraphy at Roth's collection sites, so these dates must be interpreted with caution.

#### Description

PIMUZ A/V 4165 is a nearly complete skull and mandible with all upper and lower teeth except a fragment of right I^3^ and left I_3_. Postcranial elements include all metapodials, carpals, tarsals, radio-ulnae, humeri, and tibiae as well as the left femur, a damaged left scapula, and most phalanges. The axial skeleton (pelvis, vertebrae, and ribs) and the patellae are absent.

### Skull and mandible

The skull dimensions are similar to those of *Lama guanicoe*. Length from the posterior edge of the occipital condyle to the anterior end of the rostrum is 31.5 cm (tip broken) and the width is 13.7 cm measured at the greatest breadth of the skull. The skull displays a high degree of flexion at the boundary between the basicranium and palate. The angle of flexion is 14°—it is among the highest in *Lama*, according to data provided by Webb (1965). As in all camelids, the rostrum becomes extremely tapered, starting anterior to the orbits.

**Fig. 10:**
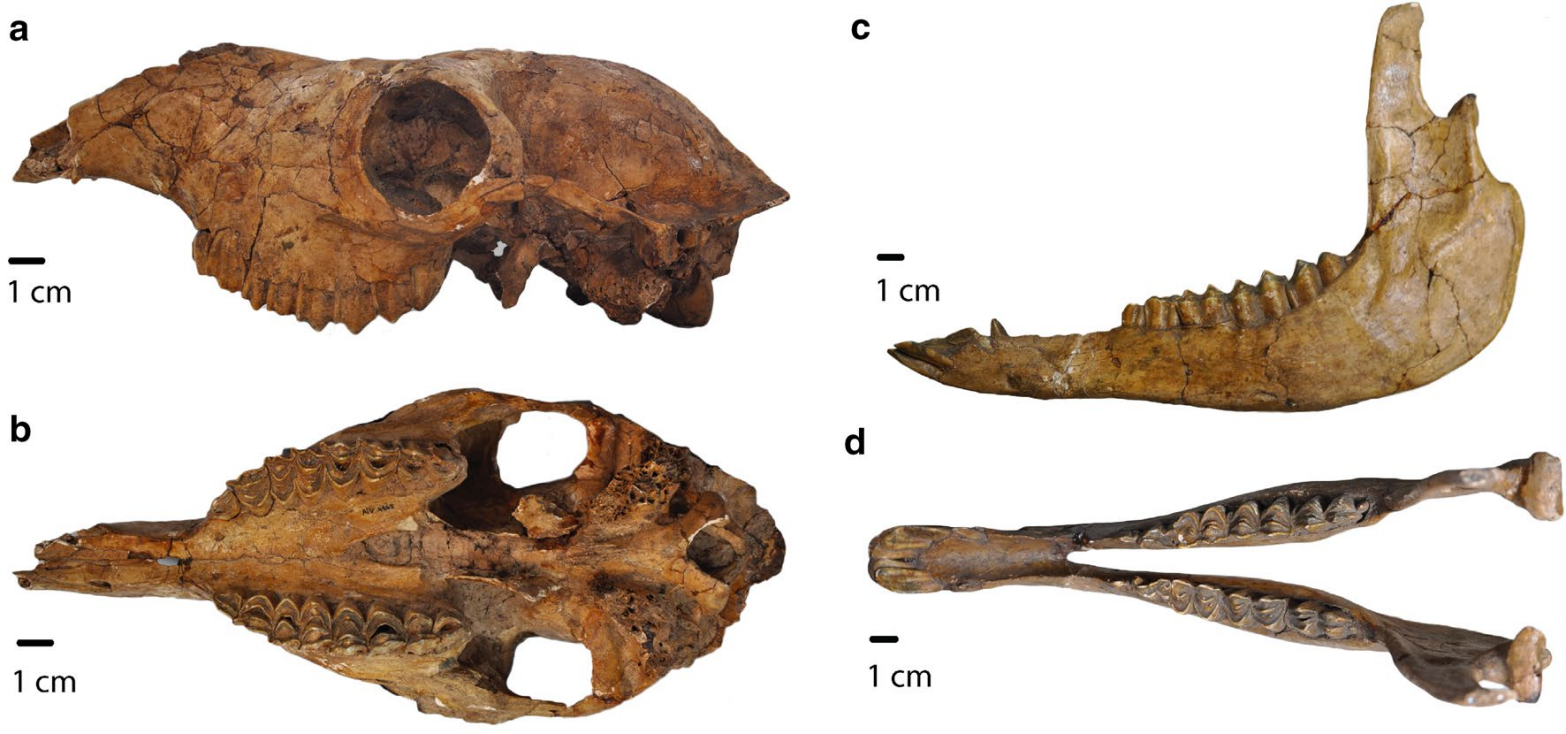
PIMUZ A/V 4165. **a** Skull lateral view. **b** Skull occlusal view. **c** Mandible lateral view. **d** Mandible occlusal view

The skull displays many morphological characters consistent with South American camelids. The zygomatic arch is sigmoid in lateral view, and the maxillary fossa is absent. The nasal bones are strongly retracted, and the anterior edge of the choanae is rounded and situated anteriorly at the level of M^2^. In more basal camelids outside of the Lamini clade, such as *Poebrotherium*, the nasal bones are longer and extend closer to the anterior edge of the premaxillae, resulting in a smaller opening. In these earlier camelids, the anterior edge of the choanae tends to be more posteriorly positioned and “V-shaped” (Fig. 10).

### Teeth

The dental formula is I1/3 C1/1 P2/2 M3/3, with premolars in the third and fourth positions. Among Lamini, this dental formula is also found in *L. guanicoe* and *H. paradoxa*. PIMUZ A/V 4165 retains the P_3_, which is absent in other camelids, such as *Camelops.* “Llama buttresses” (protostylids and parastylids) are well developed. Upper canines and incisors are small, compared to the great size which they reach in some males of extant South American camelids.

Anterior stylids (protostylids and parastylids) on M_3_ are prominent, and the anterior fossette on M_1_ is still present, making this specimen correspond to the “wear stage 3” of Breyer’s classification (Breyer 1977). The molar lophs and lophids are rounded in occlusal view. “Ribs” (labial styles) on the upper molars are well developed and P_4_ is triangular, with a fossetid on the distal lobe.

### Limbs

Compared to data on South American camelids from Scherer (2009, p. 163–166, figs. 18–19, p. 68–70), limb bones of this specimen are longer than in *Vicugna vicugna*. Most limb bones are also longer than *Lama guanicoe*, except its metacarpals which fall in the upper values of *L. guanicoe* (measurements of PIMUZ A/V 4165 in Additional file 1)*.* Compared to *Palaeolama* and *Hemiauchenia*, metapodial lengths overlap with *Palaeolama* and are smaller than in *Hemiauchenia*. Tibiae and radio-ulnae lengths both overlap with *H. paradoxa *and *P. mirifica,* and, respectively, with *P. major* and *P. weddelli*. Stylopodials are shorter than in *Palaeolama* and *Hemiauchenia*.

Metacarpals are more gracile than in *Palaeolama*. Metatarsal gracility falls within the lower values of *Palaeolama major*. Metacarpals and metatarsals are more robust than in *Vicugna* and more in the range of *Lama, H. paradoxa,* and *H. macrocephala*.

Metacarpals and metatarsals are of comparable length, with the metacarpals slightly longer than the metatarsals. In *H. paradoxa*, *H. macrocephala, P. major, P. mirifica, L. castelnaudi, L. guanicoe, *and* V. provicugna* metacarpals are longer than metatarsals or of comparable length (char. 27 in Scherer (2013)). In *H. edensis*, *P. weddelli*, and *V. vicugna,* metacarpals are shorter than the metatarsals.

**Fig. 11:**
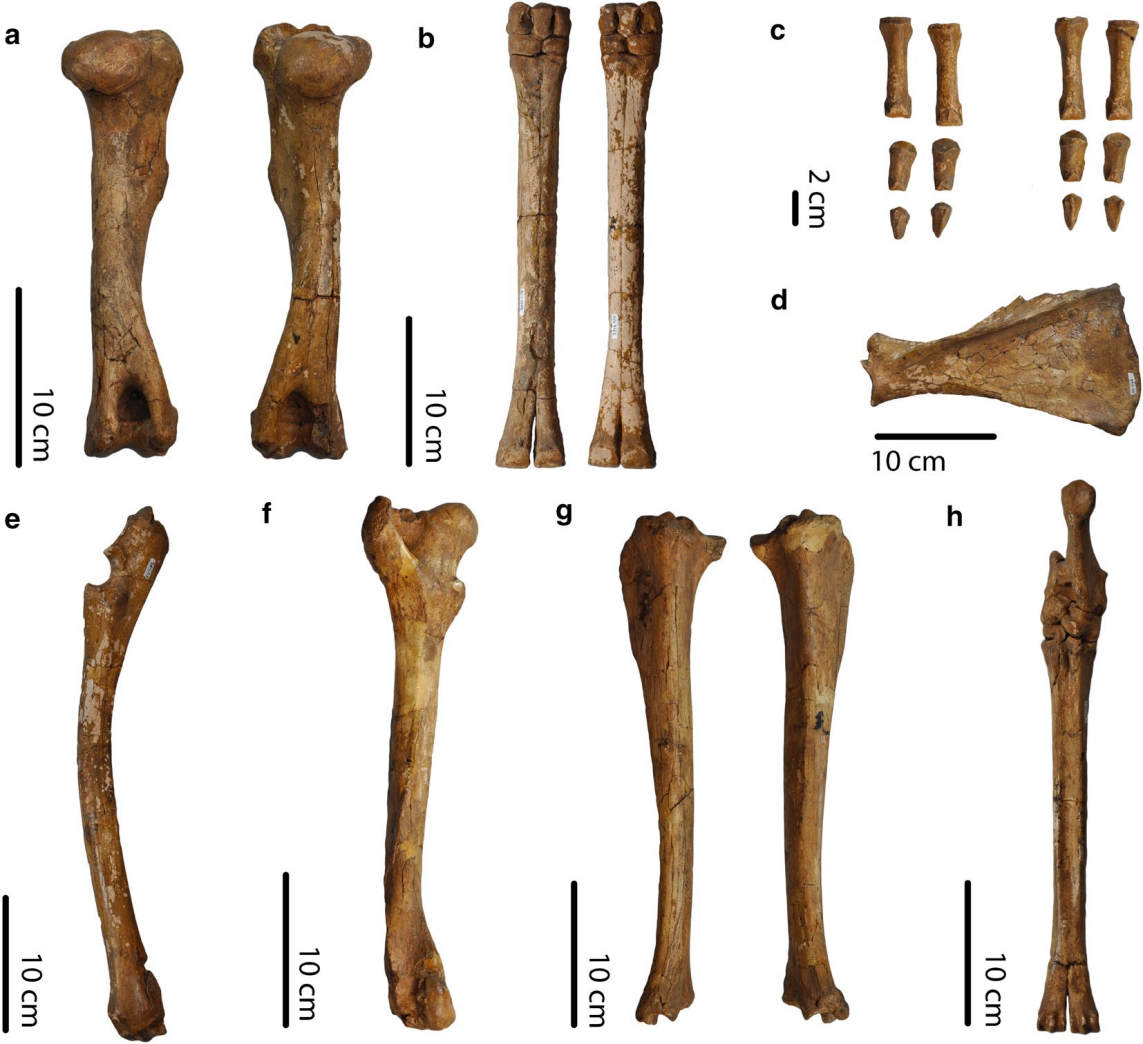
PIMUZ A/V 4165. **a** Left and right humeri, dorsal view. **b** Left and right metacarpals and carpals, ventral view. **c** Phalanges, ventral view. **d** Left scapula, lateral view. **e** Left radio-ulna, lateral view. **f** Left femur, dorsal view. **g** Right and left tibiae**,** dorsal view. **h** Right metatarsal and tarsals, dorsal view

**Fig. 12:**
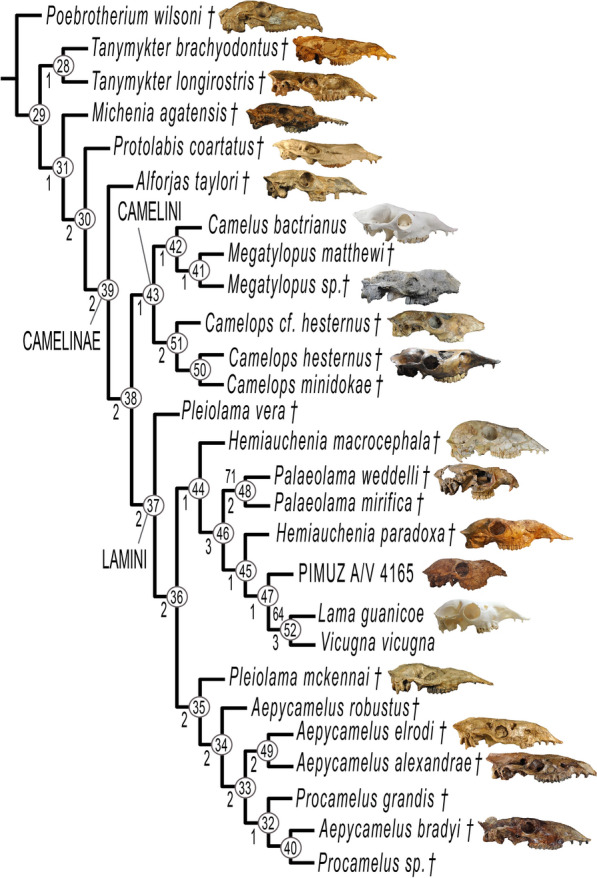
Most parsimonious tree (TL = 149 steps, CI = 0.423, RI = 0.675, RC = 0.286). Numbers in circles indicate node numbers. Numbers below branches indicate Bremer supports. Only clade 48 and clade 52 have a Bootstrap value over 50%, respectively, at 71% and 64% (see Additional file 2, for information on the skulls, pictures not to scale)

In the hind limbs, metatarsals are smaller than the tibiae and femora. Femora are shorter than the tibiae, as in *V. vicugna*, *H. paradoxa,* and *H. macrocephala* (char. 26 in Scherer (2013)). In the forelimbs, humeri are the shortest bones, and radio-ulnae are the longest. This is in contrast to some Lamini, including extant forms and *Palaeolama,* whose humeri are longer or of comparable length than their metacarpals (char. 28 in Scherer (2013)) (Figs. 11, 12).

## Results

The cladistic analysis resulted in one most parsimonious tree (Fig. 12) with length 149 (CI = 0.423, RI = 0.675, RC = 0.286). Synapomorphies are listed in Additional file 6. Both Bootstrap values and Bremer supports are low overall.

PIMUZ A/V 4165 is positioned within South American Lamini and is the sister taxon to the clade formed by *Lama guanicoe* and *Vicugna vicugna* (node 52). The synapomorphy that holds these three taxa together at node 47 is a small postglenoid process (char. 35). PIMUZ A/V 4165 has a single autapomorphy: a lower canine that is close to I_3_ (< 1 cm) (char. 7).

Well-developed protostylids and parastylids (“llama buttresses”) (char. 15) are the single synapomorphy of the clade formed by *H. paradoxa*, PIMUZ A/V 4165*, L. guanicoe,* and *V. vicugna* (node 45)*. Hemiauchenia* appears as polyphyletic. *Palaeolama* (node 48) is monophyletic; its synapomorphies are a quadrangular P_4_ with fossetids in the mesial and distal lobes (char. 14), and triangular labial lophids (char. 16)*.* The clade formed by *L. guanicoe* and *V. vicugna* (node 52) is held together by the absence of P^3^ and P_3_ (chars. 12 and 13), and the foramen ovale and median lacerate foramen are completely separated by a thin spine of the alisphenoid (char. 33). The confluence of these foramina is an autapomorphy of *L. guanicoe*.

Two synapomorphies support the monophyly of Lamini (node 37): the anterior end of the nasals arched in the transverse section (char. 23) and the anterior edge of the choanae positioned at the level of M^2^ or between M^2^ and M^1^ (char. 29). An important split within the Lamini (node 36) separates two clades: one formed by North American taxa from the Miocene (node 35), and the other formed mostly by South American taxa and two North American ones (*H. macrocephala* and *P. mirifica*) from the Pliocene–Pleistocene to the present (node 44).

*Pleiolama* is not monophyletic, appearing at two different positions within Lamini: *P. vera* is the basal-most Lamini taxon (node 37), while *P. mckennai* is the most basal taxon in the clade formed mostly by North American Lamini from the Miocene (node 35). The genera *Aepycamelus* and *Procamelus* are, respectively, polyphyletic and paraphyletic. *A. elrodi* and *A. alexandrae* are held together by four synapomorphies (node 49); *A. bradyi* forms a clade with *Procamelus sp.* (node 40) and *A. robustus* is more basal (node 34). *Procamelus* also appears in two different places, but close to each other: nodes 32 and 40.

*Camelops* (node 51) is monophyletic and placed within the Camelini (node 43). This genus is united by the absence of P^1^ (char. 8), anterior edge of the choanae U-shaped (char. 30), ethmoid foramen positioned in the fossa formed by a division of the lateral pterygoid muscle (char. 32), and the ovale and median lacerate foramina are completely separated by a thin spine of the alisphenoid (char. 33).

Camelinae (node 39) are monophyletic with three synapomorphies supporting the group: caniniform P^1^ and P_1_ (char. 8 and 9), and absence of P^2^ (char. 10). *Alforjas taylori* is a basal Camelinae, sister taxon to the clade formed by the ‘tribes’ Camelini and Lamini. Our definitions of Camelini and Lamini differ from those of Honey et al. (1998). We interpret the first major division within the Camelinae (node 38) as the split between these two clades.

The three synapomorphies uniting the Camelini (node 43) are the absence of P_3_ (char. 13), well-developed upper molar labial styles (“ribs”) (char. 17), and a lacrimal vacuity bordered by four bones, with the lacrimal border greatly reduced and the frontal border greatly enlarged (char. 25). The lacrimal vacuity bordered by two bones, frontal and maxillary, is an autapomorphy of *Camelus bactrianus*. *Megatylopus* and *Camelus bactrianus* (node 42) are united by a single synapomorphy: the absence of the premaxillary notch (char. 22).

The Protolabinae (composed of *Tanymykter*, *Michenia,* and *Protolabis*) are paraphyletic in our results. The genus *Tanymykter* (node 28) is monophyletic and supported by two synapomorphies.

## Discussion

Most of the terminal taxa were scored on the skull of a single specimen; therefore, our results could be altered by a larger sampling for each species and the addition of postcranial characters.

PIMUZ A/V 4165 appears most closely related to *L. guanicoe* and *V. vicugna* (node 47), to the exclusion of *H. paradoxa*. We found the postglenoid process (char. 35) of *H. paradoxa* to be large, whereas a small postglenoid process unites PIMUZ A/V 4165 with *L. guanicoe* and *V. vicugna*. A lower canine closer to I_3_ (< 1 cm) is the autapomorphy for PIMUZ A/V 4165. In *H. paradoxa, L. guanicoe,* and *V. vicugna,* the lower canine is positioned far from I_3_ (*≥ *1 cm) (char. 7).

We found important differences between the postcranials of PIMUZ A/V 4165 and those of *L. guanicoe* and *V. vicugna*. Among these, PIMUZ A/V 4165 has longer limb bones than *V. vicugna* and longer or among the largest sizes of *L. guanicoe* (Scherer 2009). In *V. vicugna*, metacarpals are shorter than metatarsals (Scherer 2013), whereas in PIMUZ A/V 4165, metacarpals are slightly longer than metatarsals. Metacarpals are longer than the humeri in PIMUZ A/V 4165, in contrast to *L. guanicoe* whose metacarpals are shorter than the humeri (Scherer 2009, p. 201), and *V. vicugna* whose metacarpals and humeri lengths are comparable (Scherer 2009, p. 232).

The postcranial characters observable on PIMUZ A/V 4165 are most similar to *H. paradoxa* in Scherer’s (2013) matrix (see Additional file 7). For example, as in PIMUZ A/V 4165, metacarpals are longer than humeri in *H. paradoxa*. However, an analysis using Scherer’s (2013) matrix results in a polytomy with PIMUZ A/V 4165 within the Lamini (see Additional file 7). Furthermore, compared to data from Scherer (2009), PIMUZ A/V 4165 has shorter metatarsals and humeri than *H. paradoxa*. Although the parameters of our study cannot confirm it, the fact that the morphology of PIMUZ A/V 4165 is not consistent with any single species could indicate that it belongs to a new species.

The monophyly of the Protolabinae recovered by Honey and Taylor (1978), Harrison (1985), Honey et al. (1998) and Scherer (2013) is not supported here. The species previously assigned to this group display much morphological disparity. For example, *Michenia agatensis* and *Tanymykter* retain I^1^ and I^2^ (chars. 1 and 2), while *Protolabis coartatus* has lost these teeth. We found these absences (chars. 1 and 2) to be synapomorphies uniting *P. coartatus* to the Camelinae (node 30), whereas in Scherer (2013), these were synapomorphies in her definition of the Camelinae. Here, the caniniform P_1_ and P^1^ (chars. 8 and 9) and the absence of P^2^ (char. 10) are also synapomorphies of the Camelinae (node 39). Of these, the synapomorphies of characters 8 and 9 are in agreement with Honey et al. (1998), and the synapomorphy of character 10 is in agreement with Harrison (1979).

Our phylogeny places *Alforjas taylori* in the most basal position among the Camelinae—this is in significant contrast to previous phylogenies. Harrison (1979, 1985) and Scherer (2013) placed this species in a more derived position within Lamini. In Scherer (2013), this was partly due to postcranial characters. Here and in Scherer (2013), the presence of the protostylids and parastylids (“llama buttresses”) (char. 15) on the lower molars is not a synapomorphy for the Lamini (node 37). In contrast, Harrison (1979, 1985) found this feature as a synapomorphy for the Lamini.

In Harrison (1979, 1985) and Honey et al. (1998), *Alforjas* and *Camelops* were united partly by a similar degree of hypsodonty. In these previous works and in Scherer (2013), *Alforjas* and *Camelops* were placed within Lamini and shared with them dorsally arched nasal bones in the transverse section (char. 23). We agree with this being a synapomorphy for Lamini (node 37), but we were unable to score it on *A. taylori* due to the poor preservation of the nasal bones. In *C. hesternus* and *C. cf. hesternus,* we found this feature to be dorso-ventrally flattened.

In our results, *Camelops* (node 51) is included in the Camelini (node 43). This is in agreement with recent proteomic and genetic studies which defined *Camelops* as more closely related to Camelini than to the Lamini (Buckley et al., 2019; Heintzman et al., 2015).

Harrison (1979, 1985) and Scherer (2013) found rounded canines (char. 5) to be a synapomorphy of the Camelini, whereas we found it to be a synapomorphy of the clade composed of *Aepycamelus* and *Procamelus* (node 34). *Procamelus* appeared as a basal Camelinae in Scherer (2013) and Harrison (1979). Honey et al. (1998) placed it within the Camelini. Here, as in Harrison (1985), *Procamelus* is more derived and placed within the Lamini. *Aepycamelus* is also positioned within the Lamini, as in Scherer (2013) and Honey et al. (1998). However, these genera are not monophyletic in our results, which indicates that further taxonomic revisions are necessary both taxa. We agree with observations by Honey et al. (1998) on the ambiguous status of *Aepycamelus*. We found important morphological differences between the different species of *Aepycamelus*. For instance, P^2^ (char. 10) is absent in *A. robustus*, whereas it is present in *A. alexandrae*, *A. bradyi*, and *A. elrodi*.

Scherer (2013) did not find *Hemiauchenia* to be monophyletic and we agree on this point. However, we also find a polyphyletic separation between *H. paradoxa* and *H. macrocephala*. The monophyly of *Palaeolama* (node 48) was also recovered in Scherer (2013), and in both studies, a quadrangular P_4_, with fossetids in the mesial and distal lobes (char. 14), is a synapomorphy for this genus. Here, *Palaeolama* is closely related to the genera *Hemiauchenia*, *Vicugna,* and *Lama*, whereas in Scherer (2013), *Palaeolama* was in a more basal position.

### Dental characters

When present, P^1^, P_1,_ and P_2_ (chars. 8, 9, and 11) are either premolariform or caniniform. We ordered these characters, assuming a linear evolution from a plesiomorphic premolariform state towards a caniniform state, with eventual loss in more derived taxa. At multiple points in our results, the states for these characters did not follow this linear path. For example, one of the synapomorphies at node 34 is a change from absent to a caniniform P_2_ (char. 11). Similarly, on node 33, P_2_ changes from caniniform to premolariform.

### Implications on the evolution of the Camelinae

The oldest Camelinae fossils in our analysis are those of *Aepycamelus alexandrae*, *Aepycamelus elrodi,* and *Aepycamelus robustus*, and are geologically aged to the early Barstovian, approximately (age unknown for the specimen OMNH 016,560, *A. robustus*). They all form part of the Lamini. This would imply that the appearance of the Camelinae, the divergence of Lamini from Camelini (node 38), and the main split among Lamini (node 36), all occurred before 16 Mya. Such a finding would suggest multiple ghost lineages across most of our trees. A more parsimonious conclusion would be a continuing unstable position for *Aepycamelus*, with a possible earlier divergence time for the Lamini and Camelini clades (node 38) than that estimated by Honey et al. (1998).

## Conclusion

Based on craniomandibular and dental characters, PIMUZ A/V 4165 appears more closely related to *L. guanicoe* and *V. vicugna*. However, there are important differences between the postcranials of *L. guanicoe*, *V. vicugna,* and this specimen. Although observable postcranial characters on PIMUZ A/V 4165 coincide mostly with *H. paradoxa* (Scherer 2013), their affinity was not supported in our analysis based on that matrix. We found differences in the length of some limb bones of PIMUZ A/V 4165 and *H. paradoxa*. Therefore, we suggest that PIMUZ A/V 4165 could be considered as a new species. Future systematic studies of this group with a larger sample will be able to test our results**.**

We propose several new craniomandibular and dental characters and restructure some of the relationships among Camelinae. Our results do not support the monophyly of the *Protolabinae, Aepycamelus*, *Procamelus*, *Pleiolama*, or *Hemiauchenia*. The monophyly of the *Camelinae*, *Camelops*, and *Palaeolama* are supported. We hypothesize that *Aepycamelus* and *Procamelus* form part of the Lamini and that *Camelops* is a member of the Camelini.

Several synapomorphies presented in earlier phylogenies were also recovered here, including the arched nasals (char. 23) for the Lamini (Harrison 1979, 1985; Honey et al. 1998; Scherer 2013), and the quadrangular P_4_ with fossetids in the mesial and distal lobes (char. 14) for *Palaeolama* (Scherer 2013)**.** We disagree, however, with the “llama buttresses” (char. 15) as a synapomorphy for the Lamini (Harrison 1979, 1985). Instead, we find this feature only in a more derived subgroup of this clade (node 45). We also propose new synapomorphies for several clades. Among these, we found the absence of the premaxillary notch (char. 22) to be a synapomorphy for the clade joining *Megatylopus* and *Camelus bactrianus* (node 42).

Our results superficially indicate a potential divergence between Camelini and Lamini clades before 16 Mya. This would imply that the Lamini and Camelini taxa were already present during the fourth radiation of the camelids (late Hemingfordian-to-early Barstovian) when Honey et al. (1998) date the appearance of Camelinae. More likely, the placement of the geologically older *Aepycamelus* taxa in a derived position is an indication of their continued ambiguous phylogenetic placement.

## Supplementary information

**Additional file 1.** Postcranial measurements of PIMUZ A/V 4165.

**Additional file 2.** List of specimens studied and their dental wear stage.

**Additional file 3.** Figures of measurements for ratio characters (chars. 44–49).

**Additional file 4.** Measurements for ratio characters.

**Additional file 5.** Character matrix.

**Additional file 6.** Synapomorphies.

**Additional file 7.** Phylogeny based on matrix by Scherer (2013) with PIMUZ A/V 4165.

## Data Availability

Data for the comparison of limb lengths, gracility and proportions used in the description of PIMUZ A/V 4165 can be found in Scherer (2009). The matrix used for the phylogeny, list of characters, measurements for characters, and the list of specimens studied are included in the Supplementary information files.

## Abbreviations

UF: University of Florida
UCMP: University of California Museum of Paleontology
AMNH: American Museum of Natural History
OMNH: Oklahoma Museum of Natural History
PIMUZ: Paläontologisches Museum der Universität Zürich
ZM: Zoologisches Museum der Universität Zürich
CM: Carnegie Museum of Natural History
MACN PV: Colección de Paleovertebrados, Museo Argentino de Ciencias Naturales “Bernardino Rivadavia”
MHNH: Muséum National d'Histoire Naturelle, Paris
