# Supplementary material for: Description of a fossil camelid from the Pleistocene of Argentina, and a cladistic analysis of the Camelinae
Source: Swiss J Palaeontol. 2020 Oct 7;139(1):8. doi: 10.1186/s13358-020-00208-6 (PMC7590954; doi:10.1186/s13358-020-00208-6)
Supplement: Supplementary file 1 — Additional file 1. Postcranial measurements of PIMUZ A/V 4165. [file 13358_2020_208_MOESM1_ESM.docx]

Description of a fossil camelid from the Pleistocene of Argentina, and a cladistic analysis of the Camelinae

Swiss Journal of Paleontology

Sinéad Lynch, Marcelo R. Sánchez-Villagra, Ana Balcarcel,

Palaeontological Institute and Museum, University of Zurich, Karl-Schmid-Strasse 4, 8006 Zurich, Switzerland

Corresponding Authors : Marcelo R. Sánchez-Villagra, m.sanchez@pim.uzh.ch ; Ana Balcarcel, ana.balcarcel@gmail.com

**Appendix 1: Postcranial measurements of PIMUZ A/V 4165**

| Humerus | | Radio-ulna | | Metacarpal | |
| --- | --- | --- | --- | --- | --- |
| TL | PL | TL | PL | TL | PL |
| 255 | 76 | 407 | 51 | 288 | 42 |
| Femur | | Tibia | | Metatarsal | |
| TL | PL | TL | PL | TL | PL |
| 346 | 79 | 358 | 69 | 280 | 40 |

Notes

- Measurements in millimeters
- TL = total length ; PL = proximal length
- For figures on these measurements see p.68-69, Scherer [(](https://www.zotero.org/google-docs/?7LzKRw)2009)
