# Supplementary material for: Description of a fossil camelid from the Pleistocene of Argentina, and a cladistic analysis of the Camelinae
Source: Swiss J Palaeontol. 2020 Oct 7;139(1):8. doi: 10.1186/s13358-020-00208-6 (PMC7590954; doi:10.1186/s13358-020-00208-6)
Supplement: Supplementary file 2 — Additional file 2. List of specimens studied and their dental wear stage. [file 13358_2020_208_MOESM2_ESM.docx]

Description of a fossil camelid from the Pleistocene of Argentina, and a cladistic analysis of the Camelinae

Swiss Journal of Paleontology

Sinéad Lynch, Marcelo R. Sánchez-Villagra, Ana Balcarcel

Palaeontological Institute and Museum, University of Zurich, Karl-Schmid-Strasse 4, 8006 Zurich, Switzerland

Corresponding Authors : Marcelo R. Sánchez-Villagra, m.sanchez@pim.uzh.ch ; Ana Balcarcel, ana.balcarcel@gmail.com

**Appendix 2: List of specimens studied and their dental wear stage**

| **Coll.** | **Species** | **Number** | **Material** | **Location** | **Age** | **Wear stage** |
| --- | --- | --- | --- | --- | --- | --- |
| **UF** | *Hemiauchenia macrocephala* | UF 205750 | Skull; Mandible; Postcranials | Lecanto 2A | Late Pleistocene | WS3 |
|  | *Palaeolama mirifica* | UF 89540 | Mandible | Leisey Shell Pit 1A, Hillsborough Co., Florida | Irgvingtonian, Pleistocene early | WS3 |
|  | *Palaeolama mirifica* | UF 89548 | Mandible | Leisey Shell Pit 1A, Hillsborough Co., Florida | Irgvingtonian, Pleistocene early | WS3 |
|  | *Palaeolama mirifica* | UF 64233 | Mandible | Leisey Shell Pit 1A, Hillsborough Co., Florida | Irgvingtonian, Pleistocene early | WS3 |
|  | *Palaeolama mirifica* | UF 89548 | Mandible | Leisey Shell Pit 1A, Hillsborough Co., Florida | Irgvingtonian, Pleistocene early | WS3 |
|  | *Palaeolama mirifica* | UF 64231 | Mandible | Leisey Shell Pit 1A, Hillsborough Co., Florida | Irgvingtonian, Pleistocene early | WS3 |
|  | *Palaeolama mirifica* | UF 81407 | Mandible | Leisey Shell Pit 1A, Hillsborough Co., Florida | Irgvingtonian, Pleistocene early | WS3 |
| **Coll.** | **Species** | **Number** | **Material** | **Location** | **Age** | **Wear stage** |
| **UF** | *Palaeolama mirifica* | UF 67077 | Maxilla | Leisey Shell Pit 1A, Hillsborough Co., Florida | Irgvingtonian, Pleistocene early | NA |
|  | *Palaeolama mirifica* | UF 82839 | Maxilla | Leisey Shell Pit 1A, Hillsborough Co., Florida | Irgvingtonian, Pleistocene early | NA |
|  | *Palaeolama mirifica* | UF 84882 | Mandible | Leisey Shell Pit 1A, Hillsborough Co., Florida | Irgvingtonian, Pleistocene early | WS4 |
|  | *Palaeolama mirifica* | UF 81742 | Mandible | Leisey Shell Pit 1A, Hillsborough Co., Florida | Irgvingtonian, Pleistocene early | NA |
| **AMNH** | *Alforjas taylori* (type specie) | AMNH FM 40821 (holotype) | Skull | Edson Quarry, Sherman County, Kansas | Late Hemphillian | NA |
|  | *Alforjas taylori* (type specie) | AMNH FM 40809 | Mandible | Edson Quarry, Sherman County, Kansas | Late Hemphillian | WS5 |
|  | *Alforjas taylori* (type specie) | AMNH FM 40815 | Mandible | Edson Quarry, Sherman County, Kansas | Late Hemphillian | WS5 |
|  | *Pleiolama mckennai*  (type specie) | AMNH FM 25078 (holotype) | Skull | Kat Quarry, Cherry County, Nebraska | Late Clarendonian | NA |
|  | *Pleiolama mckennai*  (type specie) | AMNH FM 33473 | Mandible | MacAdams Quarry, Donley county Texas | Late Clarendonian | WS3 |
|  | *Pleiolama vera* | AMNH FM 24670 | Skull | Edson Quarry, Sherman County, Kansas | Late Hemphillian | NA |
|  | *Pleiolama vera* | AMNH FM 24672 | Mandible | Edson Quarry, Sherman County, Kansas | Late Hemphillian | WS4 |
| **Coll.** | **Species** | **Number** | **Material** | **Location** | **Age** | **Wear stage** |
| **AMNH** | *Michenia agatensis* | AMNH FM 14255 (Genotype) | Skull; Mandible; Postcranials | Agate area, Sioux County, Nebraska | Marsland Formation (Upper Harrison) | WS5 |
|  | *Protolabis coartatus* | AMNH FM 73438 | Skull; Mandible | Deep Spring Quarry, Yavapai County, Arizona | Milk creek formation (Clarendonian) | WS5 |
|  | *Protolabis coartatus* | AMNH FM 73377 | Skull | Deep Spring Quarry, Yavapai County, Arizona | Milk creek formation (Clarendonian) | NA |
|  | *Protolabis coartatus* | AMNH FM 73309 | Mandible | Deep Spring Quarry, Yavapai County, Arizona | Milk creek formation (Clarendonian) | WS5 |
|  | *Tanymykter brachyodontus* | AMNH FM 36594 | Skull; Mandible; Postcranials | Lusk Area, Goshen county, Wyoming | Early Hemingfordian | WS4 |
|  | *Tanymykter brachyodontus* | AMNH FM 36591 | Skull; Postcranials | Lusk Area, Goshen county, Wyoming | Early Hemingfordian | NA |
|  | *Tanymykter brachyodontus* | AMNH FM 36542 | Skull; Postcranials | Lusk Area, Goshen county, Wyoming | Early Hemingfordian | NA |
|  | *Poebrotherium wilsoni* | AMNH FM 47130 | Poscranials; Mandible; Skull | Nebraska | Late chadronian-Early Orellan | WS5 |
| **OMNH** | *Aepycamelus robustus* | OMNH 016560 | Skull; Mandible; Postcranials | Roger Mills County, Oklahoma | Ogallala Fm., Age unknown | NA |
|  | *Procamelus sp.* | OMNH 79684 | Skull; Mandible; Postcranials | Ellis County, Oklahoma | Ogallala Fm., Age unknown | NA |
| **UCMP** | *Camelops cf. hesternus* | UCMP 29716 | Skull | Hetch Hetchy Tunnel, San Joaquin County, California | Rancholabrean | NA |
| **Coll.** | **Species** | **Number** | **Material** | **Location** | **Age** | **Wear stage** |
| **UCMP** | *Camelops hesternus* | UCMP 20040 | Skull; Mandible | Rancho La Brea 6, Los Angeles County, California | Rancholabrean | WS2 |
|  | *Aepycamelus alexandrae* | UCMP 26015 (holotype) | Skull; Mandible; Postcranials | Mourning's North, San Bernardino County, California | Barstovian | WS5 |
|  | *Aepycamelus bradyi* | UCMP 38668 (holotype) | Skull | Brady Pocket 1, Churchill County, Nevada | Clarendonian | NA |
|  | *Camelops minidokae* | UCMP 38446 | Skull | Irvington 2, Alameda County, California | Irvingtonian | NA |
|  | *Camelops minidokae* | UCMP 38448 | Mandible | Irvington 2, Alameda County, California | Irvingtonian | WS3 |
|  | *Megatylopus sp.* | UCMP 69464 | Skull; Postcranials | Horse Quarry 2, Twin Falls County, Idaho | Blancan | NA |
|  | *Megatylopus matthewi* | UCMP 31100 | Skull; Postcranials | Coffee Ranch Quarry 2, Hemphill County, Texas | Hemphillian | NA |
|  | *Procamelus grandis* | UCMP 32864 | Skull; Mandible; Postcranials | Big Spring Canyon General, Bennett County, South Dakota | Clarendonian | WS4 |
| **CM** | *(Procamelus elrodi)*  *Aepycamelus elrodi*  as per [Honey (1998](https://www.zotero.org/google-docs/?8lJ87m)) | CM 777 (holotype) | Skull; Mandible | Lower Madison Valley, Montana | Barstovian | WS5 |
| **Coll.** | **Species** | **Number** | **Material** | **Location** | **Age** | **Wear stage** |
| **CM** | *(Oxydactylus longirostris)*  *Tanymykter longirostris*  as per [Honey (2007](https://www.zotero.org/google-docs/?0hBEUj)) | CM 2498 (holotype) | Skull; Mandible; Postcranials | Whistle Creek, Nebraska | Harrisonian | WS5 |
| **UZH** | Indet. | PIMUZ A/V 4165 | Skull; Mandible; Postcranials | Barranca del Parana, San Nicolas, Argentina | Ensanadan | WS3 |
|  | *Vicugna vicugna* | ZM 17969 | Skull; Mandible | Zoo Zürich | Recent | WS3 |
|  | *Vicugna vicugna* | ZM 17620 | Skull; Mandible | Zoo Zürich | Recent | NA |
|  | *Vicugna vicugna* | ZM 18087 | Skull; Mandible | Zoo Zürich | Recent | WS3 |
|  | *Vicugna vicugna* | ZM 17630 | Skull; Mandible | Zoo Zürich | Recent | WS3 |
|  | *Vicugna vicugna* | ZM 17955 | Skull; Mandible | Zoo Zürich | Recent | WS5 |
|  | *Lama guanicoe* | ZM 17209 | Skull; Mandible | Zoo Zürich | Recent | NA |
|  | *Lama guanicoe* | ZM 17967 | Skull; Mandible | Zoo Zürich | Recent | WS4 |
|  | *Camelus bactrianus* | ZM 17970 | Skull; Mandible | Zoo Zürich | Recent | WS4 |
|  | *Camelus bactrianus* | ZM 17685 | Skull; Mandible | Zoo Zürich | Recent | WS3 |
| **MNHN** | *(Palaeolama reissi)*  *Palaeolama weddelli*  as per [Webb (1974](https://www.zotero.org/google-docs/?fC4JPh)) | MNHN.F.PUN1 | Skull; Mandible; Postcranials | Ecuador, Chimborazo, Punin | Pleistocene | WS3 |
| **Coll.** | **Species** | **Number** | **Material** | **Location** | **Age** | **Wear stage** |
| **MACN PV** | *Hemiauchenia paradoxa* | MACN PV 11072 | Skull; Mandible | Rio Paraná, Juan Ortiz | Lujanian ? | WS3 |

Notes : Wear stage following [Breyer (1977)](https://www.zotero.org/google-docs/?W5ZQep), not indicated if only upper dentition present. The catalog species identification of some specimens is in parenthesis. It is followed by a recombination, which is used in this analysis.
