# Supplementary material for: Description of a fossil camelid from the Pleistocene of Argentina, and a cladistic analysis of the Camelinae
Source: Swiss J Palaeontol. 2020 Oct 7;139(1):8. doi: 10.1186/s13358-020-00208-6 (PMC7590954; doi:10.1186/s13358-020-00208-6)
Supplement: Supplementary file 3 — Additional file 3. Figures of measurements for ratio characters (chars. 44–49). [file 13358_2020_208_MOESM3_ESM.docx]

Description of a fossil camelid from the Pleistocene of Argentina, and a cladistic analysis of the Camelinae

Swiss Journal of Paleontology

Sinéad Lynch, Marcelo R. Sánchez-Villagra, Ana Balcarcel

Palaeontological Institute and Museum, University of Zurich, Karl-Schmid-Strasse 4, 8006 Zurich, Switzerland

Corresponding Authors : Marcelo R. Sánchez-Villagra, m.sanchez@pim.uzh.ch ; Ana Balcarcel, ana.balcarcel@gmail.com

**Appendix 3: Figures of measurements for ratio characters (chars. 44 - 49)**

**
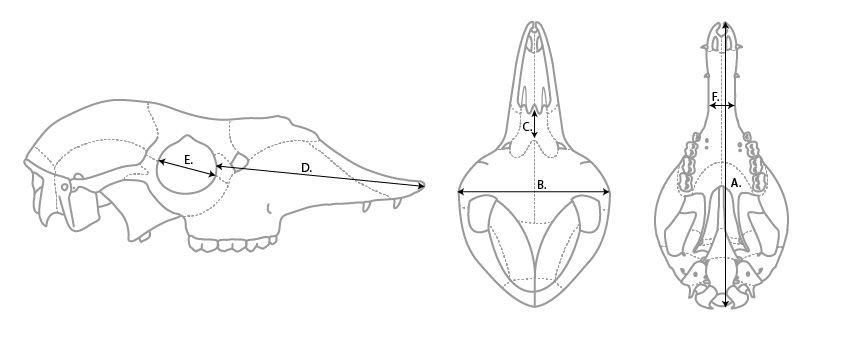
**

Schematic drawing of a skull of *Lama guanicoe* (from left to right: lateral view, dorsal view, occlusal view)

1. Length of the skull *(from the most anterior end of the rostrum, to the back of the condyle)*
2. Width of the skull *(maximum width on postorbital bars)*
3. Length of the internasal suture *(from the most anterior point to the most posterior point of the internasal suture)*
4. Rostrum length *(from the most anterior end of the rostrum to its closest point on the border of the orbit)*
5. Width of the orbit *(from the lacrimo-jugal suture on the anterior border of the orbit to the suture between the frontal and jugal bones on the posterior border of the orbit)*
6. Minimum postcanine width *(narrowest width of the rostrum in ventral view )*


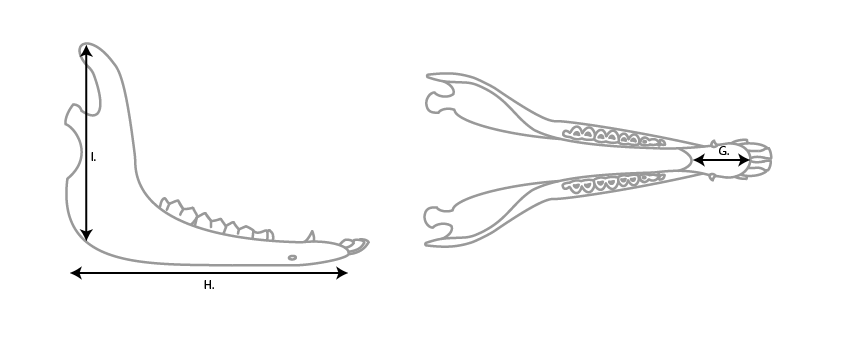


Schematic drawing of a mandible of *Lama guanicoe* (from left to right: lateral view, occlusal view)

1. Length of the symphysis *(from the most anterior point to the most posterior point of the symphysis)*
2. Length of the mandible *(from the most anterior of the symphysis to the most posterior end of the angular process)*
3. Height of the mandible *(from the highest point on the coronoid process to the ventral border of the mandible)*
