# Supplementary material for: Description of a fossil camelid from the Pleistocene of Argentina, and a cladistic analysis of the Camelinae
Source: Swiss J Palaeontol. 2020 Oct 7;139(1):8. doi: 10.1186/s13358-020-00208-6 (PMC7590954; doi:10.1186/s13358-020-00208-6)
Supplement: Supplementary file 4 — Additional file 4. Measurements for ratio characters. [file 13358_2020_208_MOESM4_ESM.docx]

Description of a fossil camelid from the Pleistocene of Argentina, and a cladistic analysis of the Camelinae

Swiss Journal of Paleontology

Sinéad Lynch, Marcelo R. Sánchez-Villagra, Ana Balcarcel

Palaeontological Institute and Museum, University of Zurich, Karl-Schmid-Strasse 4, 8006 Zurich, Switzerland

Corresponding Authors : Marcelo R. Sánchez-Villagra, m.sanchez@pim.uzh.ch ; Ana Balcarcel, ana.balcarcel@gmail.com

**Appendix 4: Measurements for ratios characters**

| **Species** | **Number** | **Sex** | **Age** | **Length of the skull** | **Width of the skull** | **Length of internasal suture** | **Rostrum length** |
| --- | --- | --- | --- | --- | --- | --- | --- |
| *H. macrocephala* | UF 205750 | NA | NA | 396 | 138 | NA | 181 |
| *P. mirifica* | UF 81407 | NA | NA | NA | NA | NA | NA |
| *A. taylori*  (type specie) | AMNH FM 40821 (holotype) | NA | NA | 393 | 168 | 84 | 232 |
| *A. taylori*  (type specie) | AMNH FM 40809 | NA | NA | NA | NA | NA | NA |
| *A. taylori*  (type specie) | AMNH FM 40815 | NA | NA | NA | NA | NA | NA |
| *P. mckennai*  (type specie) | AMNH FM 25078 (holotype) | NA | NA | 370 | NA | NA | 211 |
| *P. mckennai*  (type specie) | AMNH FM 33473 | NA | NA | NA | NA | NA | NA |
| *P. vera* | AMNH FM 24670 | NA | NA | NA | 167 | 72 | NA |
| *M. agatensis* | AMNH FM 14255 (genotype) | NA | NA | NA | 109 | NA | NA |
| *P. coartatus* | AMNH FM 73438 | NA | NA | 340 | 137 | 85 | 195 |
| *P. coartatus* | AMNH FM 73377 | NA | NA | 335 | 145 | 74 | 201 |
| **Species** | **Number** | **Sex** | **Age** | **Length of the skull** | **Width of the skull** | **Length of internasal suture** | **Rostrum length** |
| *P. coartatus* | AMNH FM 73309 | NA | NA | NA | NA | NA | NA |
| *T. brachyodontus* | AMNH FM 36594 | NA | NA | 282 | NA | 79 | 150 |
| *P. wilsoni* | AMNH FM 47130 | NA | NA | 180 | 66 | 67 | 102 |
| *A. robustus* | OMNH 016560 | NA | NA | 445 | 173 | 111 | 260 |
| *Procamelus sp.* | OMNH 79684 | NA | NA | 373 | 154 | 68 | 236 |
| *C. cf. hesternus* | UCMP 29716 | NA | NA | NA | 220 | NA | NA |
| *A. alexandrae* | UCMP 26015 (holotype) | NA | NA | 360 | 180 | 56 | 213 |
| *A. bradyi* | UCMP 38668 (holotype) | NA | NA | 518 | 204 | NA | 298 |
| *C. minidokae* | UCMP 38448 | NA | NA | NA | NA | NA | NA |
| *Megatylopus sp.* | UCMP 69464 | NA | NA | NA | 238 | 108 | NA |
| *M. matthewi* | UCMP 31100 | NA | NA | NA | 212 | NA | NA |
| *P. grandis* | UCMP 32864 | NA | NA | 419 | NA | NA | 232 |
| *A. elrodi* | CM 777 (holotype) | NA | NA | 426 | 175 | 65 | 239 |
| *T. longirostris* | CM 2498 (holotype) | NA | NA | 388 | 142 | 103 | 228 |
| Indet. | PIMUZ A/V 4165 | NA | NA | NA | 137 | NA | NA |
| *L. guanicoe* | ZM 17209 | F | 20y | 290 | 152 | 51 | 158 |
| *L. guanicoe* | ZM 17967 | F | 14y | 314 | 147 | 59 | 192 |
| *V. vicugna* | ZM 17969 | F | 6y | 234 | 121 | 28 | 129 |
| **Species** | **Number** | **Sex** | **Age** | **Length of the skull** | **Width of the skull** | **Length of internasal suture** | **Rostrum length** |
| *V. vicugna* | ZM 17620 | F | 15y | 233 | 121 | 23 | 127 |
| *V. vicugna* | ZM 18087 | F | 13y | 226 | 111 | 28 | 117 |
| *V. vicugna* | ZM 17630 | F | 6y | 225 | 115 | 25 | 120 |
| *V. vicugna* | ZM 17955 | F | 20y | 243 | 118 | 26 | 133 |
| *C. bactrianus* | ZM 17970 | F | 15y | 496 | 261 | 53 | 247 |
| *C. bactrianus* | ZM 17685 | F | 13y | 508 | 262 | 88 | 274 |

| **Species** | **Number** | **Width of orbit** | **Minimum postcanine width** | **Length of symphysis** | **Length of mandible** | **Height of mandible** |
| --- | --- | --- | --- | --- | --- | --- |
| *H. macrocephala* | UF 205750 | 46 | 13 | 78 | 317 | 195 |
| *P. mirifica* | UF 81407 | NA | NA | 78 | 300 | NA |
| *A. taylori*  (type specie) | AMNH FM 40821 (holotype) | 60 | 18 | NA | NA | NA |
| *A. taylori*  (type specie) | AMNH FM 40809 | NA | NA | 55 | 312 | NA |
| *A. taylori*  (type specie) | AMNH FM 40815 | NA | NA | 70 | 326 | 172 |
| *P. mckennai*  (type specie) | AMNH FM 25078 (holotype) | 52 | 21 | NA | NA | NA |
| *P. mckennai*  (type specie) | AMNH FM 33473 | NA | NA | 52 | 252 | 157 |
| *P. vera* | AMNH FM 24670 | 53 | 15 | NA | NA | NA |
| *M. agatensis* | AMNH FM 14255 (genotype) | 37 | 13 | NA | 217 | NA |
| *P. coartatus* | AMNH FM 73438 | 50 | 6 | 55 | 265 | NA |
| *P. coartatus* | AMNH FM 73377 | 43 | 6 | NA | NA | NA |
| *P. coartatus* | AMNH FM 73309 | NA | NA | 66 | 309 | 138 |
| *T. brachyodontus* | AMNH FM 36594 | 37 | 19 | 58 | 231 | 111 |
| *P. wilsoni* | AMNH FM 47130 | 27 | 14 | 43 | 149 | NA |
| *A. robustus* | OMNH 016560 | 45 | NA | NA | 382 | NA |
| *Procamelus sp.* | OMNH 79684 | 51 | NA | NA | 329 | 182 |
| *C. cf. hesternus* | UCMP 29716 | 68 | 29 | NA | NA | NA |
| **Species** | **Number** | **Width of the orbit** | **Minimum postcanine width** | **Length of symphisis** | **Length of mandible** | **Height of mandible** |
| *A. alexandrae* | UCMP 26015 (holotype) | 48 | 35 | 66 | 288 | NA |
| *A. bradyi* | UCMP 38668 (holotype) | 65 | 34 | NA | NA | NA |
| *C. minidokae* | UCMP 38448 | NA | NA | 83 | 374 | NA |
| *Megatylopus sp.* | UCMP 69464 | 64 | 26 | NA | NA | 268 |
| *M. matthewi* | UCMP 31100 | 54 | 30 | NA | NA | NA |
| *P. grandis* | UCMP 32864 | 53 | 25 | 83 | 351 | 218 |
| *A. elrodi* | CM 777 (holotype) | 49 | 34 | 91 | 371 | NA |
| *T. longirostris* | CM 2498 (holotype) | 49 | 21 | 75 | 347 | 161 |
|  | PIMUZ A/V 4165 | 51 | 15 | 73 | 276 | 170 |
| *L. guanicoe* | ZM 17209 | 49 | 15 | 62 | 239 | 156 |
| *L. guanicoe* | ZM 17967 | 47 | 14 | 76 | 262 | 155 |
| *V. vicugna* | ZM 17969 | 43 | 15 | 49 | 181 | 134 |
| *V. vicugna* | ZM 17620 | 45 | 14 | 52 | 186 | 127 |
| *V. vicugna* | ZM 18087 | 45 | 11 | 48 | 178 | 125 |
| *V. vicugna* | ZM 17630 | 43 | 11 | 46 | 176 | 132 |
| *V. vicugna* | ZM 17955 | 43 | 12 | 54 | 192 | 138 |
| *C. bactrianus* | ZM 17970 | 66 | 38 | 122 | 430 | 236 |
| *C. bactrianus* | ZM 17685 | 63 | 38 | 125 | 436 | 247 |

Notes:

- Measurements in millimeters
- Measurements are not present for all specimens in Appendix 3 (because of poor preservation or because I did not have direct access to them)
