# Supplementary material for: Description of a fossil camelid from the Pleistocene of Argentina, and a cladistic analysis of the Camelinae
Source: Swiss J Palaeontol. 2020 Oct 7;139(1):8. doi: 10.1186/s13358-020-00208-6 (PMC7590954; doi:10.1186/s13358-020-00208-6)
Supplement: Supplementary file 5 — Additional file 5. Character matrix. [file 13358_2020_208_MOESM5_ESM.docx]

Description of a fossil camelid from the Pleistocene of Argentina, and a cladistic analysis of the Camelinae

Swiss Journal of Paleontology

Sinéad Lynch, Marcelo R. Sánchez-Villagra, Ana Balcarcel

Palaeontological Institute and Museum, University of Zurich, Karl-Schmid-Strasse 4, 8006 Zurich, Switzerland

Corresponding Authors : Marcelo R. Sánchez-Villagra, m.sanchez@pim.uzh.ch ; Ana Balcarcel, ana.balcarcel@gmail.com

*Poebrotherium wilsoni* 000000000000000000000000000?000?000?000000000000?

*Tanymykter longirostris* 00000100?000000100?0?0?0000???0?0110?010101011111

*Protolabis coartatus* 110000000020000010000001221?000??01?10a?01101a311

*Tanymykter brachyodontus* 000001000000000100010000000?a00?00100000100111?11

*Michenia agatensis* 000011000000000100??00??000?101??11???00021???2??

*Procamelus grandis* ??000101?00000000???????????101??11?11000211???10

*Procamelus sp.* 11??010110000????0??1000321??????????10??21021??1

*Megatylopus sp.* ?????????1?0?00010???101401?101??01??????21??????

*Megatylopus matthewi* ?????????1?0????10?????140??1?1??????????????????

*Hemiauchenia macrocephala* 1100110111200000111??1???2??211??11100001211?1210

*Hemiauchenia paradoxa* 110011122120001011?1??1??20?211???1???0002102000?

PIMUZ A/V 4165 110011022120001011????1??20?211?01010100021???200

*Palaeolama mirifica* ??001?122120010111????????????1???????00021??????

*Palaeolama weddelli* ????11122120010111???0?1?2??211??1???1??020??????

*Alforjas taylori* 110011011120000000?01??0?0??000??01?1000121010211

*Pleiolama mckennai* 1100110111200001001?1011010?211?011?01000210?0010

*Pleiolama vera* ??0010011120000001??101?00?02010??????0002????2??

*Aepycamelus robustus* 110?01011110?????????01?210???????????1??20011???

*Aepycamelus alexandrae* 110?00111000000000??00?1310?1100011?01001?102101?

*Aepycamelus bradyi* 11??01?1?0?0????0010101??2??1????1?01??????0?10??

*Aepycamelus elrodi* 1100011110000?????1100?1310?0?0??110010012002101?

*Camelops minidokae* ??001?0?2?2?100011??????????1?????????01121????1?

*Camelops hesternus* 11001112212010001110000040001111111?10011210202??

*Camelops cf. hesternus* ???????2?1?0?000?????000400?11011111?0????????1??

*Vicugna vicugna* 111111122121101011111011120021a011010000021120200

*Lama guanicoe* 1100111221211010111110111200110021010100021a20200

*Camelus batrianus* 11000101112010001110a10152a11a1000101010121121101
