# Supplementary material for: Description of a fossil camelid from the Pleistocene of Argentina, and a cladistic analysis of the Camelinae
Source: Swiss J Palaeontol. 2020 Oct 7;139(1):8. doi: 10.1186/s13358-020-00208-6 (PMC7590954; doi:10.1186/s13358-020-00208-6)
Supplement: Supplementary file 6 — Additional file 6. Synapomorphies. [file 13358_2020_208_MOESM6_ESM.docx]

Description of a fossil camelid from the Pleistocene of Argentina, and a cladistic analysis of the Camelinae

Swiss Journal of Paleontology

Sinéad Lynch, Marcelo R. Sánchez-Villagra, Ana Balcarcel

Palaeontological Institute and Museum, University of Zurich, Karl-Schmid-Strasse 4, 8006 Zurich, Switzerland

Corresponding Authors : Marcelo R. Sánchez-Villagra, m.sanchez@pim.uzh.ch ; Ana Balcarcel, ana.balcarcel@gmail.com

**Appendix 6: Synapomorphies**

| *Poebrotherium wilsoni:*  No autapomorphies  *Tanymykter longirostris:*  Char. 34: 0 --> 1  Char. 39: 0 --> 1  *Protolabis coartatus:*  Char. 6: 1 --> 0  Char. 17: 0 --> 1  Char. 25: 0 --> 2  Char. 26: 0 --> 2  Char. 27: 0 --> 1  Char. 42: 2 --> 1  Char. 47: 2 --> 3  *Tanymykter brachyodontus:*  Char. 20: 0 --> 1  Char. 44: 0 --> 1  *Michenia agatensis:*  Char. 29: 0 --> 1  Char. 31: 0 --> 1  Char. 34: 0 --> 1  *Procamelus grandis:*  Char. 44: 0 --> 1  *Procamelus sp.:*  Char. 23: 1 --> 0  *Megatylopus sp.:*  No autapomorphies  *Megatylopus matthewi:*  No autapomorphies  *Hemiauchenia macrocephala:*  Char. 22: 0 --> 1  Char. 41: 0 --> 1  Char. 46: 0 --> 1  *Hemiauchenia paradoxa:*  Char. 47: 2 --> 0  PIMUZ A/V 4165:  Char. 7: 1 --> 0 | *Palaeolama mirifica:*  No autapomorphies  *Palaeolama weddeli:*  Char. 43: 1 --> 0  *Alforjas taylori:*  No autapomorphies  *Pleiolama mckennai:*  Char. 16: 0 --> 1  *Pleiolama vera:*  Char. 6: 1 --> 0  *Aepycamelus robustus:*  Char. 39: 0 --> 1  Char. 43: 1 --> 0  Char. 45: 2 --> 1  *Aepycamelus alexandrae:*  Char. 6: 1 --> 0  *Aepycamelus bradyi:*  No autapomorphies  *Aepycamelus elrodi:*  Char. 29: 1 --> 0  Char. 43: 1 --> 0  *Camelops minidokae:*  No autapomorphies  *Camelops hesternus:*  Char. 7: 0 --> 1  *Camelops cf. hesternus:*  Char. 31: 1 --> 0  *Vicugna vicugna:*  Char. 3: 0 --> 1  Char. 4: 0 --> 1  Char. 38: 1 --> 0  *Lama guanicoe:*  Char. 29: 2 --> 1  Char. 33: 1 --> 2  *Camelus batrianus:*  Char. 25: 4 --> 5  Char. 26: 0 --> 2 | Node 28:  Char. 41: 0 --> 1  Char. 46: 0 --> 1  Node 29:  No synapomorphies  Node 30:  Char. 1: 0 --> 1  Char. 2: 0 --> 1  Char. 11: 0 --> 2  Node 31:  Char. 42: 0 --> 2  Char. 47: 1 --> 2  Node 32:  Char. 37: 0 --> 1  Node 33:  Char. 10: 1 --> 0  Char. 11: 1 --> 0  Node 34:  Char. 5: 1 --> 0  Char. 11: 2 --> 1  Char. 46: 0 --> 1  Node 35:  Char. 18: 1 --> 0  Char. 47: 2 --> 0  Node 36:  Char. 26: 0 --> 1  Char. 30: 0 --> 1  Node 37:  Char. 23: 0 --> 1  Char. 29: 1 --> 2  Node 38:  Char. 18: 0 --> 1  Char. 29: 0 --> 1  Char. 31: 0 --> 1  Char. 45: 1 --> 2  Node 39:  Char. 8: 0 --> 1  Char. 9: 0 --> 1  Char. 10: 0 --> 1 | Node 40:  No synapomorphies  Node 41:  Char. 18: 1 --> 0  Node 42:  Char. 22: 0 --> 1  Node 43:  Char. 13: 0 --> 1  Char. 17: 0 --> 1  Char. 25: 0 --> 4  Node 44:  Char. 17: 0 --> 1  Char. 26: 1 --> 2  Char. 36: 0 --> 1  Node 45:  Char. 15: 0 --> 1  Node 46:  Char. 7: 0 --> 1  Char. 8: 1 --> 2  Char. 9: 1 --> 2  Node 47:  Char. 35: 1 --> 0  Node 48:  Char. 14: 0 --> 1  Char. 16: 0 --> 1  Node 49:  Char. 7: 0 --> 1  Char. 21: 1 --> 0  Char. 31: 1 --> 0  Char. 41: 0 --> 1  Node 50:  No synapomorphies  Node 51:  Char. 8: 1 --> 2  Char. 30: 0 --> 1  Char. 32: 0 --> 1  Char. 33: 0 --> 1  Node 52:  Char. 12: 0 --> 1  Char. 13: 0 --> 1  Char. 33: 0 --> 1 |
| --- | --- | --- | --- |
