# Supplementary material for: Description of a fossil camelid from the Pleistocene of Argentina, and a cladistic analysis of the Camelinae
Source: Swiss J Palaeontol. 2020 Oct 7;139(1):8. doi: 10.1186/s13358-020-00208-6 (PMC7590954; doi:10.1186/s13358-020-00208-6)
Supplement: Supplementary file 7 — Additional file 7. Phylogeny based on matrix by Scherer (2013) with PIMUZ A/V 4165. [file 13358_2020_208_MOESM7_ESM.docx]

Description of a fossil camelid from the Pleistocene of Argentina, and a cladistic analysis of the Camelinae

Swiss Journal of Paleontology

Sinéad Lynch, Marcelo R. Sánchez-Villagra, Ana Balcarcel

Palaeontological Institute and Museum, University of Zurich, Karl-Schmid-Strasse 4, 8006 Zurich, Switzerland

Corresponding Authors : Marcelo R. Sánchez-Villagra, m.sanchez@pim.uzh.ch ; Ana Balcarcel, ana.balcarcel@gmail.com

**Appendix 7: Phylogeny based on matrix by Scherer (2013) with PIMUZ A/V 4165**


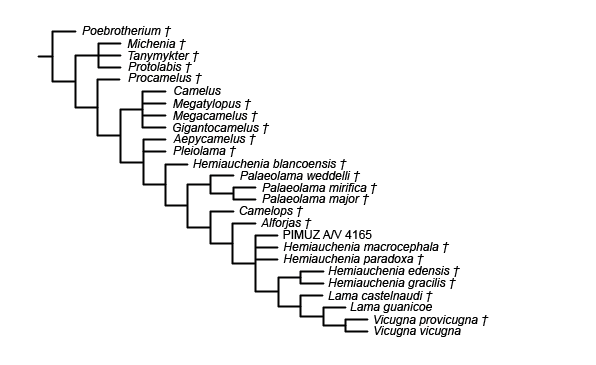


Strict consensus (L = 82 ; CI = 0.439 ; RI = 0.763 ; RC = 0.335) of 12 most parsimonious trees. Analysis based on matrix by Scherer (2013), conducted in TNT (Goloboff and Catalano 2016) using a traditional search with 6000 replicates and 10 trees saved per replications. Scoring for PIMUZ A/V 4165: *?110012?01100111100010?1111111?1?00*. The scoring for all other taxa follows Scherer (2013).
